# Activated monocytes and markers of inflammation in newly diagnosed multiple sclerosis

**DOI:** 10.1111/imcb.12337

**Published:** 2020-05-05

**Authors:** Mikkel Carstensen, Tove Christensen, Morten Stilund, Holger J Møller, Eva L Petersen, Thor Petersen

**Affiliations:** ^1^ Department of Biomedicine Aarhus University Skou Building, Høegh‐Guldbergsgade 10 DK‐8000 Aarhus C Denmark; ^2^ Department of Neurology Aarhus University Hospital Palle Juul‐Jensens Boulevard 165 DK‐8200 Aarhus N Denmark; ^3^ Department of Clinical Biochemistry Aarhus University Hospital Palle Juul‐Jensens Boulevard 99 DK‐8200 Aarhus N Denmark

**Keywords:** cytokines, HERVs, inflammation, monocyte subsets, monocytes, multiple sclerosis

## Abstract

In multiple sclerosis (MS), the inflammation and demyelination of the central nervous system (CNS) develop in distinct ways. This makes diagnosing patients difficult, imperative to initiating early and proper treatment. Several common features exist, among them a profound infiltration of monocytes into the CNS mediating demyelination and tissue destruction. In the periphery, monocytes are divided into three subsets depending on expression of CD14 and CD16, representing different stages of activation and differentiation. To investigate their involvement in MS, peripheral blood mononuclear cells (PBMCs) from 61 patients with incipient, untreated MS and 22 symptomatic control (SC) patients as well as 6 patients with radiologically isolated syndrome (RIS) were characterized *ex vivo*. In addition, paired serum and cerebrospinal fluid (CSF) samples were analyzed with a panel of biomarkers. In PBMC samples, we demonstrate decreased levels of nonclassical monocytes with a concomitant significant decrease of human endogenous retrovirus (HERV) H3 envelope epitopes on this monocyte subset compared with SC and RIS. The observed HERV expression is present on nonclassical monocytes irrespective of MS and thus presumably a result of the inflammatory activation. For the other surface markers analyzed, we found significantly decreased expression between classical and nonclassical monocytes. In matched samples of CSF a highly significant increase in levels of soluble markers of activation and inflammation is shown, and notably this is not the case for the serum samples. Of the soluble markers investigated, interleukin (IL)‐12/IL‐23p40 had the highest discriminatory power in differentiating patients with MS from SC and RIS, almost comparable to the immunoglobulin G index.

## Graphical abstract

Characterizing monocyte subsets in newly diagnosed multiple sclerosis (MS) contributes valuable insight into disease mechanisms and potential therapeutic targets. We present evidence of inflammation‐driven activation of monocytes in peripheral blood as well as detailed analysis of biomarker panels capable of differentiating MS from symptomatic controls.

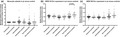

## Introduction

Multiple factors influence the initiation and progression of the neuroinflammatory disease multiple sclerosis (MS). Intense research efforts have so far not succeeded in identifying a single, defined biomarker for the disease.^1^, ^2^ Studies of families with MS have defined genetic variants that may account for up to one‐third of the inherent susceptibility,^3^ but environmental factors such as Epstein–Barr virus infection or smoking still account for the majority of risk factors for MS.^4^ Without a predominant exogenous risk factor, it thus remains an open question whether MS is triggered in the periphery or in the central nervous system (CNS).^5^ Despite the considerable heterogeneity of MS, several disease subtypes have been recognized, and generally, patients experience either relapsing‐remitting (RRMS) or progressive (PMS) disease courses. Patients who do not have spreading of symptoms in both time and space are diagnosed with clinically isolated syndrome (CIS), of which most (eight out of ten) eventually progress to MS.^6^ Newly revised guidelines for MS diagnostics^7^ establish the diagnosis of MS if the first symptoms with objective neurological findings and typical MS abnormalities on magnetic resonance imaging (MRI) are combined with a positive test for oligoclonal bands or an increased immunoglobulin G (IgG) index. This emphasizes the importance of biomarkers in MS diagnostics. A number of potential biomarkers have thus been investigated in various body fluids, but given the complex nature of the disease, only a few candidates have been considered as contributions to the diagnostic criteria of MS.^8^

In addition to soluble markers, several key surface receptors involved in immune regulation are shed from the surfaces of immune cells as a result of inflammatory stimuli.^9^, ^10^, ^11^ A combination of surface‐bound and secreted molecules may therefore have potential as diagnostic and prognostic markers of disease and progression.^12^, ^13^ Multifactorial processes of inflammation and neurodegeneration can thus be accounted for and biomarker panels presently hold the best promise for earlier diagnosis and improved prognostics.^14^ In biomarker panels, an individual marker may not achieve sufficiently high sensitivity and specificity, but combined with other markers may contribute positively to the overall diagnostic and/or prognostic value of the test.^15^

Historically, the main focus in the pathogenesis of MS has been on autoreactive T cells, but substantial evidence now also implies other cell types (e.g. peripheral monocytes) as prominent cell types early in disease,^16^ mediating both proinflammatory and anti‐inflammatory responses.^17^, ^18^ In peripheral blood, monocytes are divided into three subsets, each performing different functions by differential expression of antigens and cytokines,^19^, ^20^ and it has recently been established that the three subsets are a result of a gradual maturation process.^21^, ^22^ Expression levels of CD14 and CD16 are used to distinguish classical (CD14^++^CD16^−^), intermediate (CD14^++^CD16^+^) and nonclassical (CD14^+^CD16^++^) monocyte subsets,^23^ and similar subsets exist in other species, for example, in mice based on LY6C, CCR2 and CX3CR1 expression.^24^

In a number of chronic inflammatory diseases such as arthritis,^25^ coronary heart disease,^26^ MS,^27^ psoriasis^28^ or HIV encephalitis,^29^ monocytes exhibit characteristics of activation during periods of disease activity. The different pathways of monocyte activation predetermine the immune response in each individual. This may have led to the inconsistencies in attributing which subsets are proinflammatory and which ligands elicit migration toward inflammation.^19^, ^30^, ^31^, ^32^ In pathologies involving the CNS, however, it is recognized that specifically CCR1, CCR2 and CCR5 are among the most important receptors for migration.^33^ These receptors are highly expressed on classical monocytes, but evidence also indicates that intermediate and patrolling nonclassical monocytes are migrating into the CNS and promoting infiltration of other cells as well as tissue destruction.^17^

We have previously shown that in patients with MS compared with healthy controls, there is a significant increase of nonclassical monocytes with a concomitant reduction of classical monocytes.^34^ In addition, a significant reduction of several surface receptors was found on the intermediate and nonclassical monocytes, possibly because of increased shedding activity. By contrast, expression of human endogenous retrovirus (HERV) envelope (Env) epitopes, encoded by both HERV‐H/F and HERV‐W, was localized to the nonclassical monocytes. Expression of HERVs has previously been associated with neurological diseases, notably MS,^35^ and we have previously demonstrated expression of these HERV epitopes in patients with active MS^36^, ^37^ as well as an HERV‐directed humoral immune response.^38^, ^39^, ^40^ In addition to this, our previous studies of monocytes were accompanied by analysis of selected markers of inflammation and neurodegeneration in serum and cerebrospinal fluid (CSF), establishing the potential of combining several biomarkers into a single multivariable model.^15^, ^34^

To further investigate the role of monocyte subsets as well as HERVs in patients with MS, we here present further analysis of the circulating monocytes and their subsets in newly diagnosed patients with MS. In this study, we compared the newly diagnosed patients with MS to a group of patients presenting with MS‐like symptoms but without documented objective neurological findings: symptomatic controls (SCs), enrolled during the diagnostic workup.

Furthermore, as defining biomarkers for MS remains elusive, we have investigated a panel of 17 soluble biomarkers, illustrating essential components in the pathogenesis of MS focusing on inflammation and monocyte/macrophage activity (see Supplementary table 1 for a review of the selected soluble markers).

## Results

Patients enrolled in this study were undergoing diagnostic workup for MS and thus largely represent incipient MS. Patients that fulfill some, but not all of the diagnostic criteria for MS are often encountered in the clinic. Here, these patients comprise the SC group if they did not have another defined neurological disease (please refer to the Methods for further details). This is an advantage for the proficiency of the biomarker analysis, as clinically relevant biomarkers ideally should be able to distinguish these patient groups. Initially, 92 patients were enrolled during a period of 2 years (see Supplementary figure 1). Of those, one opted out and two were excluded after subsequent diagnoses of anti‐AQP4‐positive NMO and motor neuron disease, respectively. The final cohort comprises 22 patients with CIS, 33 patients with RRMS, 6 patients with PMS, 6 patients with radiologically isolated syndrome (RIS) and 22 SCs (see Supplementary table 2). The demographics of the cohort are presented in Table 1.

**Table 1:** Demographic and clinical data of patients with CIS, RRMS, PMS, RIS and SC.

Characteristics	CIS	RRMS	PMS	RIS	SC
No. of patients (*N* = 89)	*n* = 22	*n* = 33	*n* = 6	*n* = 6	*n* = 22
Gender (M/F)	(9/13)	(9/24)	(1/5)	(0/6)	(4/18)
Age^a^	38	37	52	39.5	37
(range)	(22–63)	(23–54)	(45–60)	(35–51)	(20–58)
EDSS^b^	2.0	2.5	3.0	N/A	N/A
(range)	(0–3.5)	(0–6.0)	(2.5–4.5)	N/A	N/A
IgG index	0.75	0.77	0.74	0.45	0.44
(range)	(0.47–1.72)	(0.44–2.22)	(0.56–1.28)	(0.42–0.47)	(0.39–0.56)
OCB positive	16	32	6	0	0
Progressed at follow‐up^c^	9	12	5	0	0
Medication at follow‐up^d^	11	30	0	0	0
Time since last attack (days)^e^	61	71	N/A	N/A	N/A
(range)	(7–399)	(4–307)	N/A	N/A	N/A
Follow‐up time (months)	24	28	25	24	25
(range)	(17–37)	(9–41)	(17–40)	(17–36)	(18–39)
Patients in the TNL category^f^					
0 lesions	1	0	0	0	10
1–10 lesions	14	13	2	6	0
11–20 lesions	4	9	1	0	0
>20 lesions	2	11	3	0	0
Unspecific WM lesions	1	0	0	0	12

CIS, clinically isolated syndrome; EDSS, Expanded Disability Status Scale; N/A, not applicable or available; OCB, oligoclonal bands; PMS, progressive multiple sclerosis; RIS, radiologically isolated syndrome; RRMS, relapsing‐remitting multiple sclerosis; SC, symptomatic control; WM, white matter.

^a^ Median age (in years), at the time of sampling.

^b^ Median values of EDSS, determined at diagnostic workup.

^c^ Progressed at follow‐up, estimated on the basis of a clinically defined attack or a sustained increase of more than 0.5 in the EDSS scale within a follow‐up period of 9–41 months (median = 25).

^d^ Individuals receiving immune‐modulating therapy within the follow‐up period *versus* individuals that did not.

^e^ Time since last attack: the period of time (in days) from latest attack to the sampling.

^f^ TNL, Total number of white matter lesions determined by fluid‐attenuated inversion recovery sequences on magnetic resonance imaging.

**Figure 1:**
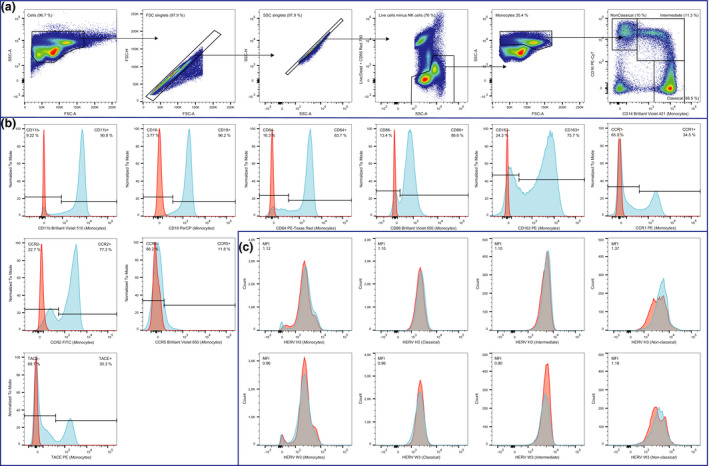
**Gating strategy used in the flow cytometric analysis. **A sample from a representative patient with RRMS was used for this figure. **(a)** From left to right: Total PBMCs (> 300,000 events) were gated according to their size and granularity in forward scatter‐area (FSC‐A) / side scatter‐area (SSC‐A); aggregated cells were removed according to forward scatter‐area (FSC‐A) / FSC‐H and side scatter‐area (SSC‐A) / SSC‐H leaving only singlets; dead cells as well as NK‐cells were removed according to staining with a LIVE/DEAD cell stain and monoclonal anti‐CD56 antibody, respectively; monocytes (> 100,000 events) were gated in forward scatter‐area (FSC‐A) / side scatter‐area (SSC‐A); and finally the three monocyte subsets (classical, intermediate, non‐classical) were gated according to their CD14/CD16 expression. **(b)** From left to right: Surface marker expression of CD11b, CD18, CD40, CD64, CD86, CD163, CCR1, CCR2, CCR5, and TACE were determined by gating on positive cells (blue). Appropriate isotype controls (red) were used to determine the unspecific antibody binding. **(c)** From left to right: Human endogenous retrovirus (HERV) expression was determined on the monocyte population, and on the three monocyte subsets (classical, intermediate, non‐classical) by incubation with sera from rabbits immunized with HERV H3 Env (top row) or HERV W3 Env (bottom row) peptide antigens (blue) as described previously ^36^ and with the appropriate control (pre‐immune sera) (red) to determine the positive populations (shaded grey area represent the overlap between the two curves).

Peripheral blood mononuclear cells (PBMCs) were isolated by density‐gradient centrifugation and subjected to flow cytometric analysis to characterize the three distinct monocyte subsets as well as expression of other markers of activation and cell signaling. The gating strategy used for the flow cytometric analyses is shown in Figure 1.

The median levels and range of all the surface markers analyzed are presented in Supplementary table 3, showing that the HERV H3 Env epitopes are expressed at a significantly lower level in patients with MS or CIS than in SC and RIS. Supplementary table 3 also shows the existing overlap in the surface marker levels for the five patient groups. A Kruskal–Wallis test with Dunn’s multiple comparisons test (α = 0.05) was used to calculate the significance of differences between groups.

**Figure 2:**
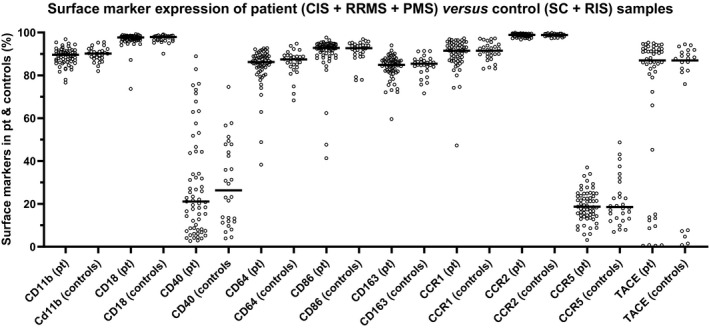
Differences in the expression of CD11b, CD18, CD40, CD64, CD86, CD163, CCR1, CCR2, CCR5 and TACE on the monocytes. The differences in expression of CD11b, CD18, CD40, CD64, CD86, CD163, CCR1, CCR2, CCR5 and TACE on the monocyte population (Monocytes, Figure 1a) from the five patient groups were determined as the percentage of positive cells. Bars represent the median of the populations. CIS, clinically isolated syndrome; PMS, progressive multiple sclerosis; Pt, patients; RIS, radiologically isolated syndrome; RRMS, relapsing‐remitting multiple sclerosis; SC, symptomatic control.

**Figure 3:**
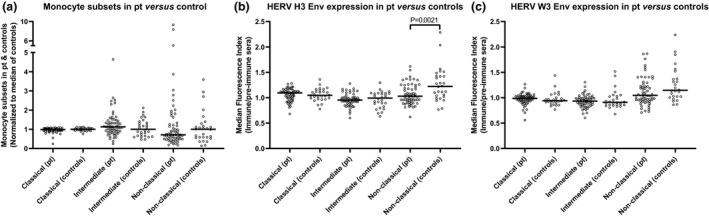
Differences in the monocyte subsets between patient groups and in the expression of HERV Env epitopes. **(a)** The differences in the three monocyte subsets (classical, intermediate and nonclassical) between patient (CIS + RRMS + PMS) and control (SC + RIS) samples, normalized to the median of the control samples for each subset; **(b)** expressions of HERV H3 Env and **(c)** HERV W3 Env on the three monocyte subsets were determined as the median fluorescence index by calculating the median fluorescence for each sample and dividing by the median fluorescence of the appropriate control (preimmune sera). Bars represent the median of the subsets and braces indicate a significant difference (Mann–Whitney *U*‐test) between the median of the patient group (*n* = 61) and the median of the control group (*n* = 28). *P*‐values are shown. Env, envelope; CIS, clinically isolated syndrome; HERV, human endogenous retrovirus; PMS, progressive multiple sclerosis; Pt, patients; RIS, radiologically isolated syndrome; RRMS, relapsing‐remitting multiple sclerosis; SC, symptomatic control.

In Supplementary table 4, the variation in cell surface marker expression between MS groups and correlations with clinical disease measures are presented. Overall, no significant differences were seen in surface marker expression between RRMS and PMS, between MS and CIS or between RIS and SC, and we thus further grouped these patients into MS + CIS and SC + RIS. Of particular interest in comparing patients with MS + CIS *versus* SC + RIS is the significant difference between HERV H3 Env epitopes on the nonclassical monocyte population, making it the only surface marker analyzed that is able to distinguish between these patient groups (Figures 2 and 3). Furthermore, no differences in surface marker expression were found in patients with MS + CIS with regard to gender distribution, oligoclonal band status, progression or medication at follow‐up.

Expression of surface markers on the three monocyte subsets is shown in Supplementary figure 2. Interestingly, the differences in surface marker expression between the monocyte subsets indicate a highly coordinated downregulation or shedding of surface receptors on the intermediate and nonclassical monocytes compared with classical monocytes, except for TACE on the intermediate subset. Mann–Whitney *U*‐tests were used to calculate the significance of differences between surface marker expression on the monocyte subsets.

**Figure 4:**
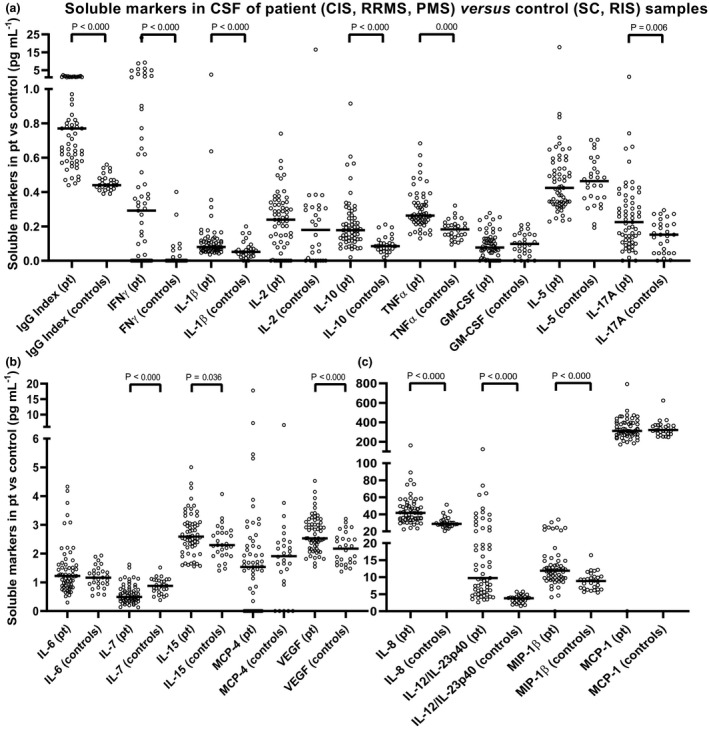
Differences in the soluble marker levels in CSF of patient (CIS + RRMS + PMS) *versus* control (SC + RIS) samples. The soluble markers were graphed in pg mL^−1^: **(a)** the markers with a median between 0 and 1 (pg mL^−1^); **(b)** the markers with a median between 0 and 6 (pg mL^−1^); **(c)** the markers with a median above 6 (pg mL^−1^). The CSF IgG index = (CSF IgG × serum albumin) × (CSF albumin × serum IgG)^−1^ is included here as the index of local IgG production and it serves as a reference biomarker. Bars represent the median of the population and braces indicate a significant difference (Mann–Whitney *U*‐test) between the median of the patient group (*n* = 61) and the median of the control group (*n* = 28). *P*‐values are shown. CIS, clinically isolated syndrome; CSF, cerebrospinal fluid; Env, envelope; GM‐CSF, granulocyte‐macrophage colony stimulating factor; IFNγ, interferon gamma; Ig, immunoglobulin; IL, interleukin; MCP‐1, monocyte chemoattractant protein; MIP‐1β, macrophage inflammatory protein‐1β; PMS, progressive multiple sclerosis; Pt, patients; RIS, radiologically isolated syndrome; RRMS, relapsing‐remitting multiple sclerosis; SC, symptomatic control; TNFα, tumor necrosis factor alpha; VEGF, vascular endothelial growth factor.

The analyses of cell surface marker expression on peripheral blood monocytes were accompanied by multiplex measurements of an extensive panel of relevant soluble markers in paired CSF and serum samples from the study cohort. Supplementary tables 5 and 6 illustrate variations in levels of soluble markers in CSF and serum, respectively, between the MS‐relevant groups as well as correlations with clinical disease measures. No distinct differences were seen for the soluble markers in CSF between RRMS and PMS, between MS and CIS or between RIS and SC, indicating the suitability of the grouping of patients into MS + CIS and SC + RIS. When comparing these patient groups, however, highly significant differences were seen in CSF for interferon‐γ, interleukin (IL)‐1β, IL‐8, IL‐10, tumor necrosis factor alpha (TNFα), IL‐7, IL‐12/IL‐23p40, IL‐17A, macrophage inflammatory protein‐1β and vascular endothelial growth factor (VEGF), indicating that such biomarkers may be used individually—or preferably in combination—to differentiate patients with MS + CIS from SC + RIS (see also Figure 4). As seen from Supplementary table 6, this is not evident for the serum samples, where only serum IL‐7 is significantly different between MS + CIS and SC + RIS. In the present study, this illustrates that the levels of biomarkers in serum were not correlated with the inflammatory processes occurring in the CNS, whereas levels of biomarkers in the CSF were highly distinct for the two groups.

Supplementary table 7 shows the median levels and range of the soluble markers analyzed in CSF and serum and the CSF‐to‐serum ratio. This table illustrates that the biomarker levels differ significantly between the MS group and the CS + RIS control group mainly in CSF and CSF‐to‐serum ratio. Serum IL‐2, serum IL‐7 and serum IL‐12/IL‐23p40 do, however, differ significantly between PMS and SC; between RRMS, PMS, RIS and SC and between PMS, RIS and SC, respectively, but only contribute little to the differences in the ratio. The results in Supplementary table 7 also illustrate the existing overlap in the soluble biomarker levels for the five patient groups.

**Figure 5:**
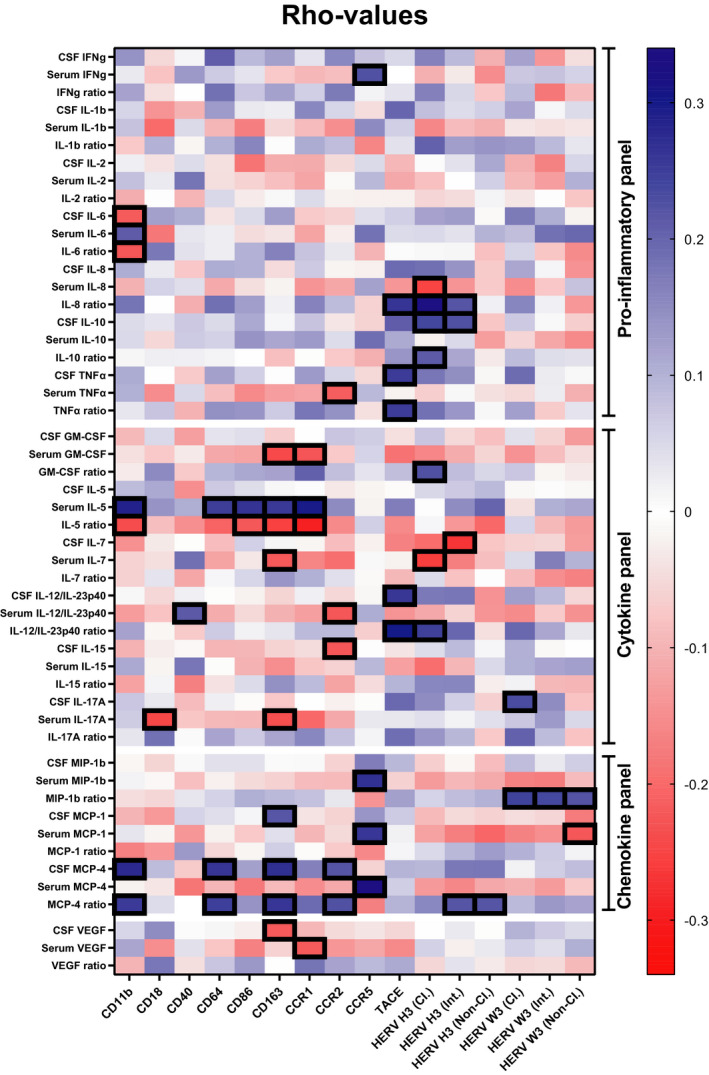
Correlations between surface and soluble markers of activation and inflammation. Rho values are graphed in a heatmap based on correlations between surface markers and soluble markers in CSF, serum and their respective ratios. Blue indicates positive correlations and red indicates negative correlations. Correlations with a significant *P*‐value are marked with a black frame. CSF, cerebrospinal fluid; GM‐CSF, granulocyte‐macrophage colony stimulating factor; IFNγ, interferon gamma; IL, interleukin; MCP‐1, monocyte chemoattractant protein; MIP‐1β, macrophage inflammatory protein‐1β; Pt, patients; TNFα, tumor necrosis factor alpha; VEGF, vascular endothelial growth factor.

To further investigate the role of the surface and soluble biomarkers, the surface and soluble markers in CSF, serum and their respective ratios were correlated using Spearman’s correlations. Figure 5 shows the rho values of the correlations; both positive (blue) and negative (red) correlations are shown. Correlations with a significant *P*‐value are marked with black frames (see Supplementary table 8 for rho and *P*‐values). Interestingly, several surface markers correlate significantly with the soluble markers and specifically surface‐bound CD163 correlates significantly with nine of the soluble markers. Of those, serum IL‐7 and CSF VEGF also differ significantly between the patients with MS + CIS and the controls SC + RIS. However, the only surface marker that differs significantly between patients with MS + CIS and SC + RIS is the HERV H3 Env on the nonclassical monocyte population, and HERV H3 Env only correlates significantly with the monocyte chemoattractant protein (MCP)‐4 ratio, which does not differ significantly between any of the five patient groups. By contrast, the expression of the receptor CCR‐5 correlates significantly with three chemotactic proteins/ligands in serum as well as the proinflammatory INFγ. Similarly, expression of TACE correlates significantly with IL‐12/IL‐23p40 and TNFα in CSF but not in serum, indicating that there are other sources of these proteins in serum, for example, tissue homing or resident leucocytes at distal sites of inflammation.

**Figure 6:**
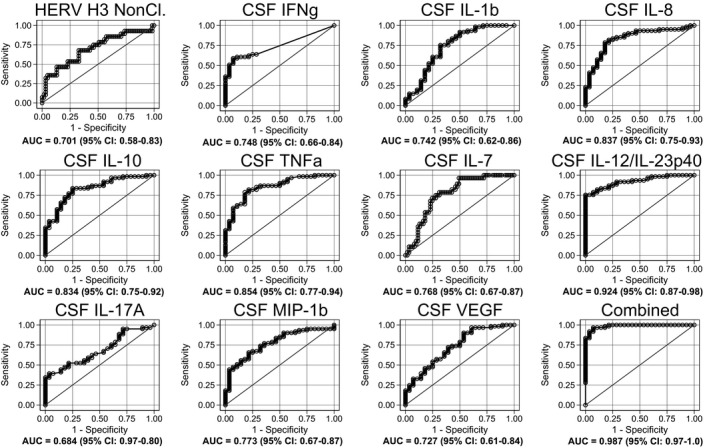
Logistic regression analyses with receiver operating characteristic curve output of surface and soluble markers. AUC, with 95% CI, is given for each parameter. The surface expression of each parameter for patients (*n* = 61) is combined as true positives and plotted against controls (*n* = 28) as true negatives. The diagonal dividing the ROC space represents the random event. In addition, a logistic regression analysis with combined parameter results was performed for “all parameters.” AUC, area under the curve; CSF, cerebrospinal fluid; HERV, human endogenous retrovirus; IFN, interferon; IL, interleukin; ROC, receiver operating characteristic; TNF, tumor necrosis factor; VEGF, vascular endothelial growth factor.

To explore the biomarker potential of the surface and soluble markers that are significantly different between patients with MS + CIS and SC + RIS, a logistic regression analysis with receiver operating characteristic curve output was performed. Figure 6 shows the receiver operating characteristic curves with area under the curve (AUC) as a measure of their respective discriminatory power. As seen from the graphs, the discriminatory power of all the markers are higher than 70%, except for IL‐17A in CSF for which this is 68.4%, and are thus fair markers of disease as defined by Xia *et al*.^14^ When all contributions are combined, the discriminatory power is 98.7% [95% confidence interval (CI) 0.97–1.0]; 6% higher than any of the individual contributions. Notably, the contributions from both TNFα and IL‐12/IL‐23p40 in CSF are higher than 85%, and in combination these amount to 94.7% (95% CI 0.90–0.99) (see Supplementary figure 3). Besides, when HERV H3 Env on the nonclassical monocyte population and IL‐12/IL‐23p40 in CSF are combined with the IgG index with a discriminatory power of 96.4% (95% CI 0.93–0.99), an AUC of 99.5% (95% CI 0.99–1.0) is obtained (see Supplementary figure 4).

Finally, to explore the potential of the investigated biomarkers to predict disease progression or if patients with MS + CIS will receive medication after diagnostic workup, a similar regression analysis was performed for the soluble markers that were statistically significant for the two parameters, respectively. Supplementary figure 5 shows the receiver operating characteristic curves for granulocyte‐macrophage colony stimulating factor and MCP‐1 in CSF as well as their combined contribution to predict disease progression. As seen from the figure, the AUCs for the three graphs are all below 68% and are thus the aforesaid are poor prognostic markers in differentiating patients with MS + CIS that have progressed from those that have not. Similarly, Supplementary figure 6 shows the receiver operating characteristic curves for IL‐2 in CSF and serum, IL‐12/IL‐23p40 in CSF as well as their combined contribution to predict if patients receive medication or not after the diagnosis. As seen from the figure, both IL‐2 in serum and IL‐12/IL‐23p40 in CSF have AUC > 70% and are thus fair markers; however, when combined with IL‐2 in CSF, the AUC is 68.8% (95% CI 0.54–0.83) and thus do not contribute positively to a combined AUC. Lastly, the IgG index had an AUC of 57.1% (95% CI 0.42–0.72) for predicting disease progression and 64.5% (95% CI 0.49–0.80) for predicting whether patients with MS + CIS receive medication and did not contribute positively to either of the prognostic assessments.

## Discussion

Characterizing monocytes and their subsets in incipient nontreated MS facilitates important insight into early mechanisms of disease, and is unaffected by disease progression or modifying treatment. In addition, the inclusion of SCs recruited during the diagnostic workup facilitates the use of any potential, highly specific biomarkers and panels hereof, directly applicable to a clinical setting.

In recent years, many new therapeutics or disease‐modifying therapies have been approved for MS therapy, either immunosuppressants or more specific antibodies targeting T and B cells in the periphery.^41^, ^42^, ^43^, ^44^ Targeting monocytes or subsets hereof may also have potential in MS therapy,^45^, ^46^ as it is indicated in animal models of MS that depletion of monocytes inhibit both initiation of disease and progression.^47^

In the present study, we have analyzed the composition of monocyte subsets and expression of markers of activation and cell signaling in incipient MS, together with a comprehensive panel of inflammation‐associated signaling molecules in paired CSF and serum samples.

In samples from patients with CIS, RRMS, PMS, RIS or SC variations in the median total number of monocytes were in general low, as were the variation of the different monocyte subsets between these patient groups, except for the nonclassical monocytes where a marked lower median was observed for patients with MS + CIS than for SC + RIS. In our previous study, we noted a marked increase in the nonclassical monocyte subset with a concomitant downregulation of the classical subset, compared with healthy controls.^34^ A detailed comparison, however, revealed a similar pattern of monocyte subsets in the two studies, with a large proportion of patients with MS + CIS having the same number of monocyte subsets as the SC + RIS or healthy controls as well as a number of significant outliers. One striking difference though was the high number of nonclassical monocytes in some of the SC and RIS patients in the current study, emphasizing that SC and RIS are indeed not comparable to healthy controls.

For the surface markers investigated, we found a significantly lower expression of HERV H3 Env epitopes on the nonclassical monocytes in patients with MS + CIS *versus* SC and MS + CIS *versus* SC + RIS. In our previous study comprising incipient and progressed patients with MS compared with a healthy control group, the numbers of nonclassical monocytes and the expression levels of HERV Env were higher for the patients with MS.^34^ Our present results on monocyte subsets are in line with a recent report^17^ which additionally found increased numbers of nonclassical monocytes in the CSF, probably indicating their migration into the CNS. Furthermore, in our previous study one‐third of the patients included were treated with immune‐modulating therapy, and it is known that nonclassical monocyte populations may increase after treatment initiation.^17^

The HERVs investigated here are associated with a number of neurologic diseases^35^, ^48^ and likely also with the inflammatory processes, and may thus be expressed because of a defective mechanism of chromatin‐dependent repression.^49^, ^50^

Interestingly, in both patients with MS + CIS and SC + RIS, we found a marked downregulation of expression of all the measured surface markers on the nonclassical monocytes compared with intermediate or classical, except for CD40 and TACE, where the intermediate monocytes have higher expression. This pattern of expression is in agreement with our previous study,^34^ and in the present study, we also show this for CD11b, CD18, CD64, CD86, CCR1 and CCR5. Surprisingly, the expression of CD40 and TACE was highest in the intermediate monocyte population, indicating that these monocytes indeed have high stimulatory and inflammatory potential.^51^, ^52^

For the soluble biomarkers investigated in CSF and serum, a highly significant difference was demonstrated between patients with MS + CIS and SC + RIS in 11 of 17 biomarkers measured in CSF. This was also evident in the CSF‐to‐serum ratios. Levels were significantly higher in MS + CIS samples, except for IL‐7, which is a hematopoietic growth factor that promotes generation and survival of lymphoid cells.^53^ In addition, patients with MS + CIS with the highest levels of, for example, INFγ tended to have high levels of most of the other biomarkers, except granulocyte‐macrophage colony stimulating factor and VEGF, for which no differences were seen (data not shown). This illustrates that multifactorial processes of immune regulation are at play in any given time point of MS disease, as most of the biomarkers have relatively short half‐lives.^54^ Interestingly, biomarker levels in serum were not directly correlated with the inflammatory process occurring in CNS, and the only significant difference found was for IL‐7, which was higher in MS sera compared with SC + RIS. Previously, IL‐7 has been shown to be lower in patients with MS, as well as the IL‐7 receptor (IL‐7Rα) expression associated with MS pathogenesis,^55^ although the mechanisms are not clear. Furthermore, that study was based on comparisons with healthy controls, which might explain the discrepancy.

In the present correlation analysis, several surface markers were significantly correlated with levels of soluble markers in both CSF and serum as well as their respective ratios. Notably, surface‐bound CD163, which correlated significantly with nine of the soluble markers, is worthy of attention, as we have previously demonstrated a significant association between CD163 and MS.^15^, ^34^ In addition, expression of CCR5 correlated significantly with three proteins for monocyte chemotaxis in serum, and it has been shown that CCR5^+^ mononuclear cells accumulates in the CNS of MS patients.^56^ Finally, expression of TACE correlated significantly with several soluble markers, notably TNFα and IL‐12/IL‐23p40 in CSF but not in serum. This is interesting, as treatment with monoclonal antibodies against TNFα in MS has shown increased disease activity,^57^, ^58^ and treatment with neutralizing antibodies toward IL‐12/IL‐23p40 can be used to treat several autoimmune diseases,^59^ but no effect was seen in MS.^60^ However, further investigations of this last treatment could be indicated in selected patients with high levels of IL‐12/IL‐23p40 in serum and comorbidity with psoriasis.

In the logistic regression analysis, the HERV H3 Env expression on the nonclassical monocytes as well as several soluble biomarkers performed well in differentiating patients with MS + CIS from HC + RIS, and a combined regression analysis of 11 biomarkers showed excellent performance with an AUC of 98.7%. It is noteworthy that IL‐12/IL‐23p40 had an excellent performance, even when used alone, with an AUC of 92.4% almost comparable to the IgG index with an AUC of 96.4%.

Notably, when IL‐12/IL‐23p40 was combined with TNFα with an AUC of 85.4%, the combined AUC was 94.7%, but when combined with HERV H3 Env on the nonclassical monocytes with an AUC of 70.1% and the IgG index, the combined AUC was 99.5%, higher than any other combination of the investigated biomarkers. A combination of a surface biomarker and two soluble biomarkers thus performs almost perfectly in differentiating patients with MS + CIS from SC + RIS. However, none of the investigated biomarkers could predict progression, or differentiate between patients with MS + CIS that received immune modulating therapy and not, emphasizing that longer follow‐up periods may be needed for some of the patients.

We have previously demonstrated that combinations of specific biomarkers enable pertinent differentiation between patients with MS + CIS and SC^15^ as well as MS + CIS and HC,^34^ illustrating that in a multifactorial diseases such as MS, the combination of multiple biomarkers into a single multivariable model provides a high level of diagnostic discrimination.^15^ Here we demonstrate an AUC of 99.5% with only three biomarkers combined, emphasizing the additional potential of especially IL‐12/IL‐23p40 as a diagnostic marker in incipient MS.

Taken together, the present results of significant changes in expressed inflammation‐related cell surface markers between the three monocyte subsets, as well as the HERV Env epitopes expressed on the nonclassical monocyte populations, are interesting. It substantiates our hypothesis that monocyte subsets recruited to plaque formations in the CNS carrying viral epitopes maybe contribute to the recurrent and/or continued inflammatory activity.^34^ We do also find these HERV epitopes expressed in RIS and SC patients, with the marked difference that these patients likely have intact blood–brain barriers and thus few nonclassical monocytes homing to the CNS.^61^

Importantly, we also present significant changes in 11 inflammation‐related signaling molecules in MS + CIS compared with SC + RIS, illustrating highly coordinated responses in patients with MS + CIS, and that some of these molecules are excellent in differentiating MS‐like disease from non‐MS.

Overall, the results indicate that clear definition of a multifactorial disease such as MS is only possible using carefully selected combinations of biomarkers and that development of a small but proficient biomarker panel is possible.

## Methods

### Ethics statement

The study was conducted in accordance with the Ethical Declaration of Helsinki and all patients gave written, informed consent. The study and the material for informed consent were approved by The Central Denmark Region Committee on Biomedical Research Ethics (journal number: 1‐10‐72‐202‐13).

### Patients and controls

Patients admitted to the MS clinic, Department of Neurology, Aarhus University Hospital, were consecutively included from June 2015 to February 2018. A full diagnostic workup included medical history, clinical examination, MRI of the entire neural axis, CSF analyses (cells, protein, IgG index) and evoked potentials as recommended.^62^ CSF and MRI examination were evaluated according to the revised MacDonald criteria^62^ and an Extended Disability Status Scale score was assessed according to Kurtzke.^63^ Patients were excluded if they had other neurologic disease, received glucocorticoids or other immune modulating treatments within the month preceding sampling. One patient was diagnosed with neuromyelitis optica and one with motor neuron disease. One patient diagnosed with MS wanted to be excluded from the study and withdrew her consent. Total number of MRI white matter lesions were registered by fluid‐attenuated inversion recovery sequences on MRI. Demographics and paraclinical findings of patients with CIS, RRMS, PMS, RIS and SCs are summarized in Table 1. A clinical description of the patients with RIS and SC is presented in Supplementary table 9.

Patients included as SC have neurological symptoms, but have no objective clinical or paraclinical findings to define a specific neurological disease. This specific definition is described in detail by Teunissen *et al.* ^64^ and they do not represent early MS.

Patients included as RIS have no neurological symptoms and are only referred to diagnostic workup owing to the presence of incidental white matter lesions in MRI suggestive of MS. Diagnostic criteria for RIS were proposed in 2009 and include the number, shape and location of the brain lesions.^65^ Lesions are ovoid and well circumscribed with a size greater than 3 mm, show dissemination in space and can be juxtaposed to the corpus callosum. Lesions should not follow a vascular distribution and do not account for any other pathologic processes.^66^, ^67^ Recently, the risk of conversion of RIS to MS was reported as depending on (1) involvement of spinal cord, (2) younger age, (3) oligoclonal bands positive and/or increased IgG index and (4) infratentorial lesions, with each factor adding to a combined risk score.^68^ The RIS patients in the present study fulfill the risk factor 2 (younger age) but none of the others. The risk of conversion for zero or one factor present is 29% over 10 years.^68^ The number of lesions for the RIS patients is included in Supplementary table 9.

Disease activity was ascribed on the basis of a new clinically defined attack or a sustained increase of more than 0.5 on the Extended Disability Status Scale within a follow‐up period of 9–41 months (median = 25). Medication at follow‐up was ascribed to those receiving any immune‐modulating therapy in the follow‐up period. In total, 89 patients agreed to participate in this study and in accordance with consensus guidelines,^69^ serum and CSF were frozen at −70°C until use, while whole PBMCs were isolated and frozen at −150°C until use.

### PBMC isolation

Each collected sample was labeled with a study ID at inclusion and all the analysis were performed blinded to the clinical status of the patients. PBMCs were isolated from heparinized whole blood using Ficoll‐Paque PLUS (Amersham Biosciences, Umeå, Sweden, Catalog number 17‐1440‐02) within a few hours after drawing. In brief, whole blood was mixed with phosphate‐buffered saline (PBS) (Sigma, Welwyn Garden City, UK, Catalog number D8537) in 1:1 and 20 mL Ficoll‐Paque was layered underneath about 25 mL of the diluted blood. The tubes were centrifuged at 750*g* for 25 min at room temperature, and PBMCs were collected from the interface layer. Then PBMCs were washed three times with 20 mL PBS and centrifuged at 450*g* for 15 min, 280*g* for 10 min and finally at 190*g* at 10 min, to remove the sugar gradient and impurities (platelets). PBMCs were frozen in Roswell Park Memorial Institute‐1640 medium (BioWhittaker, Bornem, Belgium, Catalog number BE12‐702F) supplemented with 20% (heat‐inactivated and sterile‐filtrated) human serum and 10% dimethyl sulfoxide (Sigma, Lyon, France, Catalog number D4540). Before use, each portion of frozen PBMCs was quickly thawed (37°C); washed once in 10 mL ice‐cold Roswell Park Memorial Institute‐1640 medium containing 10 mm HEPES, 0.03% w v^−1^ glutamine, 0.2 mio IU L^−1^ penicillin–streptomycin and 10% (heat‐inactivated and sterile‐filtrated) human serum and resuspended to a concentration of 40 × 10^6^ cells mL^−1^.

### FLOW analysis

Before staining PBMCs with antibodies, samples were first incubated with the amine‐reactive reagent LIVE/DEAD Fixable Near‐IR Dead Cell Stain (Life Technologies, Eugene, OR, USA, #L10119), then washed with PBS (pH 7.4) and blocked in PBS + 0.2% human serum albumin (CSL Behring, Marburg, Germany, #109697) + 100 µg mL^−1^ h‐IgG (human serum albumin; Privigen 10%, CSL Behring) + 0.09% NaAz (Ampliqon, Odense, Denmark, #AMPQ52310.0250) for 15 min at 4°C. Samples were then washed two times and labeled by incubating with a mixture of different monoclonal antibodies for 30 min in the dark at 4°C, washed two times in PBS and resuspended in 100 µL PBS + 0.2% human serum albumin + 0.09% NaAz. The following monoclonal mouse antibodies were used to stain 1 × 10^6^ PBMCs in 100 µL PBS: 5 µL anti‐CD14 Brilliant Violet 421 (BD, San Jose, CA, USA; clone MϕP9, IgG2b, #563743), 10 µL anti‐CD16 PC7 (Beckman Coulter, Chaska, MN, USA; clone 3G8, IgG1, #6607118), 10 µL anti‐CD18 Per‐CP (Abcam, Cambridge, UK; clone GRF1, IgG1 #GR3187629‐1), 5 µL anti‐CD11b BV‐510 (BioLegend, San Diego, CA, USA; clone ICRF44, IgG1, #301334), 5 µL anti‐CD40 PerCP (eBioscience, San Diego, CA, USA; clone 5C3, IgG1, #17‐0409‐42), 5 µL anti‐CD64 PE‐CF594 (BD; clone 10.1, IgG1, #565389), 5 µL anti‐CD86 BV‐650 (BD; clone 2331, IgG1, #563412), 2 µL anti‐CD163 PE (Trillium Diagnostics, Bangor, ME, USA; clone Mac2‐48, IgG1, #CD163‐48P), 5 µL anti‐CCR1 PE (BioLegend, San Diego, CA, USA; clone 5F10B29, IgG1, #362904), 5 µL anti‐CCR2 FITC (BioLegend; clone K036C2, IgG2a, #357216), 5 µL anti‐CCR5 BV‐650 (BD; clone 3A9, IgG2a, #564999) and 5 µL anti‐CD56 AF‐750 (Beckman Coulter; clone 3G8, IgG1, #B46024).

Detection of HERV H3 and HERV W3 expression was performed using polyclonal antisera as previously described.^36^ In brief, antibodies against an HERV‐H/F Env peptide epitope (HERV H3) and an HERV‐W/MSRV Env peptide epitope (HERV W3) were raised in New Zealand White rabbits, and preimmune sera were collected from all rabbits before immunization and used as background staining/control. The peptide epitopes are localized at equivalent positions in open reading frames at the respective HERV loci. Both peptides and antisera were made by Sigma Genosys (UK). Polyclonal antibody binding to target cells was visualized using 10 µL diluted goat antirabbit Alexa Flour 488 (Life Technologies, #A‐11034) for 30 min in the dark at 4°C, washed two times in PBS and resuspended in 100 µL PBS + 0.2% human serum albumin + 0.09% NaAz.

Flow cytometric analyses were performed using an LSRFortessa (BD) equipped with four lasers [a violet (405 nm), blue (488 nm), yellow (561 nm) and a red (640 nm)]. FlowJo software version 10 (FlowJo LLC, Ashland, OR, USA) was used for data analysis. More than 100 000 events were collected for further analysis. For each surface marker, all of the flow data were collected in one run. To validate the compensation matrices and gating strategy, appropriate fluorescence‐minus‐one controls were made (not shown).

### Multiplex

Multiplex electrochemiluminescence immunoassays from Meso Scale Discovery (Rockville, MD, USA) was used to measure levels of 17 selected cytokines and growth factors in paired samples of CSF and serum from all the included patients. The measured soluble factors were interferon‐γ, IL‐1β, IL‐2, IL‐6, IL‐8, IL‐10, TNFα, granulocyte‐macrophage colony stimulating factor, IL‐5, IL‐7, IL‐12/IL‐23p40, IL‐15, IL‐17A, macrophage inflammatory protein‐1β, MCP‐1, MCP‐4 and VEGF. The soluble factors can be divided into a proinflammatory panel consisting of interferon‐γ, IL‐1β, IL‐2, IL‐6, IL‐8, IL‐10 and TNFα; a cytokine panel comprising granulocyte‐macrophage colony stimulating factor, IL‐5, IL‐7, IL‐12/IL‐23p40, IL‐15 and IL‐17A; a chemokine panel consisting of macrophage inflammatory protein‐1β, MCP‐1, MCP‐4 and finally the growth factor VEGF. All factors were measured according to the manufacturer’s instructions, and concentrations were calculated by reference to a standard curve for each molecule. Standards were assayed in the same manner as the CSF and serum samples. The lower and upper limits of detection were calculated based on the concentration of signal equal to 2.5 × s.d. above the zero calibrator and below the upper plateau of the standards curve, respectively. All samples were analyzed in duplicate.

### Collection of data and statistical analysis

Personal data were stored and handled according to the Danish law. Collection of demographic and biochemical data was done using the Electronic Patient Journal (EPJ). Descriptions of MRI were conducted by a neuroradiologist and confirmed by a senior neurologist at the Department of Neurology, AUH, who viewed all scans in the IMPAX system. For data collection we used Microsoft Excel (Microsoft, Redmond, WA, USA) and all statistical analysis was performed using STATA version 15 (StataCorp LLC, College Station, TX, USA).

## Conflict of Interest

The authors declare no conflict of interest.

## Supporting information

Supplementary Material
