# Supplementary material for: Activated monocytes and markers of inflammation in newly diagnosed multiple sclerosis
Source: Immunol Cell Biol. 2020 May 5;98(7):549–62. doi: 10.1111/imcb.12337 (PMC7496724; doi:10.1111/imcb.12337)
Supplement: Supplementary file 1 — Supplementary Material [file IMCB-98-549-s001.docx]

**Supplementary information to:**

**Activated monocytes and markers of inflammation in newly diagnosed multiple sclerosis**

| Supplementary table I1. Soluble biomarkers investigated | | |
| --- | --- | --- |
| Marker | Occurrence | Action |
| IFN-γ | Produced by lymphocytes upon stimulation | Pro-inflammatory cytokine involved in numerous pathways and a potent activator of macrophages |
| IL-1β | Produced by activated monocytes/macrophages | Pro-inflammatory cytokine stimulates T cell proliferation, B cell maturation/proliferation, and fibroblast growth factor activity |
| IL-2 | Produced by T-cells | Crucial regulator of the immune system through T-cell proliferation |
| IL-6 | Secreted mainly by T cells and macrophages | Also known as B-cell stimulatory factor 2, involved in numerous biological functions including inflammation, aging, cell growth, apoptosis, and bone remodelling |
| IL-8 | Leucocytes | Pro-inflammatory cytokine involved in attraction of neutrophils, basophils, and T-cells but not monocytes. |
| IL-10 | Monocytes, lymphocytes | Downregulates the expression of pro-inflammatory Th1 cytokines, MHC class II antigens, and enhances B cell survival, proliferation, and antibody production |
| TNF-α | Monocytes, leucocytes upon stimulation | Pro-inflammatory cytokine that can induce sepsis, inflammation and can inhibit tumorigenesis and viral replication |
| GM-CSF | Macrophages, leucocytes upon stimulation | Cytokine that stimulate growth and differentiation of hematopoietic precursor cells in response to inflammatory stimuli, increase ROS |
| IL-5 | T helper cells, mast cells, eosinophils | Regulate eosinophil accumulation in tissues and induce terminal differentiation of B cells to immunoglobulin secreting cells |
| IL-7 | Stromal, dendritic, epithelial cells | hematopoietic growth factor involved in cell-cell signalling and proliferation of B cells |
| IL-12/IL-23p40 | Macrophages upon stimulation | Common subunit of IL-12 and IL-23, cytotoxic lymphocyte maturation factor that acts on T and NK cells to produce IFNγ and TNFα |
| IL-15 | Monocytes, macrophages | Stimulates the proliferation of T and NK cells, partially redundant with IL-2 |
| IL-17A | T and NK cells upon activation | Induces proinflammatory and hematopoietic cytokines in stromal cells and ICAM1 expression on fibroblasts |
| MIP-1β | Monocytes, lymphocytes | Down-regulates the expression of CCR5 and thus inhibits the CCR5-dependent signal transduction |
| MCP-1 | Monocytes, macrophages | Recruits monocytes, T cells, and basophils to sites of inflammation |
| MCP-4 | Epithelial cells, monocytes | Attracts leucocytes to sites of inflammation, induced by IL-1 and TNFα |
| VEGF | Hypoxic, cells upon activation | Stimulates the formation of new blood vessels, induced by TNFα |
| sDC163 (soluble CD163), IFNγ (interferon gamma), IL-1β (interleukin-1β), IL-2 (interleukin-2), IL-6 (interleukin-6), IL-8 (interleukin-8), IL-10 (interleukin-10), TNFα (tumor necrosis factor alpha), GM-CSF (granulocyte-macrophage colony stimulating factor), IL-5 (interleukin-5), IL-7 (interleukin-7), IL-12/IL-23p40 (interleukin-12/interleukin-23 p40), IL-15 (interleukin-15), IL-17A (interleukin-17A), MIP-1β (macrophage inflammatory protein-1β), MCP-1 (monocyte chemoattractant protein-1), MCP-4 (monocyte chemoattractant protein-4), VEGF (vascular endothelial factor). | | |

**Supplementary figure I1**

**
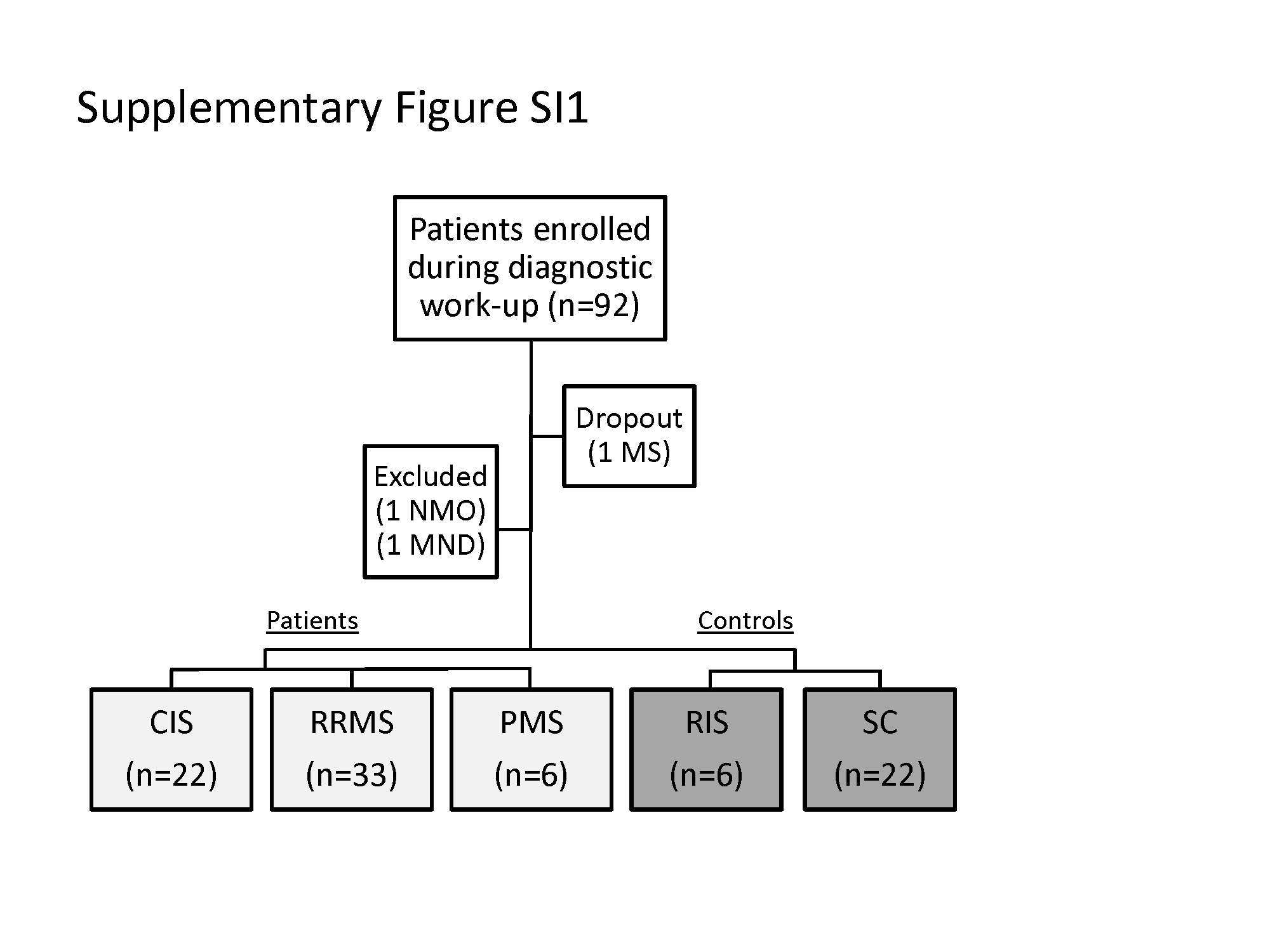
**

**Supplementary figure I1. Flowchart of the study cohort.** Ninety-two patients were included at diagnostic work-up, and one patient with MS subsequently chose to dropout. Patients were not included if they had other neurologic disease or received methylprednisolone treatment within one month prior to sampling. Two patients were excluded: one due to the diagnosis of neuromyelitis opticus (NMO), with confirmed seropositivity for anti-AQP4 autoantibodies, and one due to a motor neuron disease (MND). Patients included in the study cohort were stratified according to disease progression and the revised MacDonald criteria (Polman, 2011) and comprised the following subgroups: CIS (clinically isolated syndrome), RRMS (relapsing-remitting MS), PMS (progressive MS), RIS (radiologically isolated syndrome), and SC (symptomatic controls).

**Supplementary table I2. Basic dataset.** Excel file above (double click to activate) contains all basic data for the paper. CIS (clinically isolated syndrome), RRMS (relapsing-remitting MS), PMS (progressive MS), RIS (radiologically isolated syndrome), SC (symptomatic control), EDSS (expanded disability status scale), CSF (cerebrospinal fluid), Gender (M=male; F=female), y (years), m (months), d (days).

| **Supplementary table I3. Median levels and range of markers on the monocyte cell surface as determined by flow cytometric analyses for the five patient groups.** | | | | | | |
| --- | --- | --- | --- | --- | --- | --- |
| **Characteristics** | **CIS** | **RRMS** | **PMS** | **RIS** | **SC** | **KW p-value**  **(significance)** |
| **No. of Subjects** (total=89) | n = 22 | n = 33 | n = 6 | n = 6 | n = 22 |  |
| **No. of monocytes** (x 1000) | 109.5 | 105.8 | 149.9 | 101.9 | 123.0 | 0.58 (ns) |
| **(range)** | (30.2 – 246.6) | (49.9 – 233.5) | (91.0 – 199.4) | (69.8 – 145.2) | (44.8 – 200.7) |  |
| **CD11b** % | 88.6 | 90.1 | 87.4 | 91.3 | 89.5 | 0.28 (ns) |
| **(range)** | (76.6 - 96.9) | (81.7 - 95.4) | (83.9 - 94.9) | (88.2 - 95.5) | (82.0 - 94.1) |  |
| **CD18** % | 97.1 | 97.8 | 97.6 | 98.1 | 97.9 | 0.93 (ns) |
| **(range)** | (73.7 - 98.7) | (94.1 - 99.2) | (96.9 - 98.3) | (90.1 - 98.8) | (95.0 - 99.1) |  |
| **CD40** % | 14.3 | 23.4 | 22.7 | 17.45 | 32.6 | 0.18 (ns) |
| **(range)** | (2.87 - 76.1) | (2.68 - 88.9) | (6.07 - 71.6) | (3.89 - 31.3) | (4.45 - 74.6) |  |
| **CD64** % | 86.5 | 85.9 | 86.4 | 88.6 | 86.7 | 0.92 (ns) |
| **(range)** | (48.8 – 92.0) | (38.3 - 92.6) | (70.9 - 92.7) | (74.8 - 94.8) | (68.3 - 93.8) |  |
| **CD86** % | 92.7 | 93.1 | 93.0 | 91.7 | 93.3 | 0.97 (ns) |
| **(range)** | (47.6 - 95.6) | (41.3 - 97.7) | (82.5 - 95.0) | (77.7 - 96.9) | (77.9 - 96.1) |  |
| **CD163** % | 83.5 | 85.7 | 85.7 | 86.4 | 84.7 | 0.21 (ns) |
| **(range)** | (59.6 - 90.2) | (73.6 - 94.0) | (75.5 - 89.6) | (84.9 - 91.2) | (71.6 - 91.4) |  |
| **CCR1** % | 90.4 | 91.5 | 92.8 | 93.5 | 91.0 | 0.87 (ns) |
| **(range)** | (74.2 - 96.6) | (47.3 - 97.3) | (82.4 - 96.9) | (83.7 - 96.7) | (83.1 - 97.2) |  |
| **CCR2** % | 87.3 | 86.0 | 82.8 | 87.0 | 87.0 | 0.86 (ns) |
| **(range)** | (58.1 - 92.4) | (66.9 - 92.1) | (71.9 - 92.1) | (79.8 - 93.1) | (65.5 - 93.8) |  |
| **CCR5** % | 17.4 | 21.4 | 19.3 | 27.6 | 17.5 | 0.26 (ns) |
| **(range)** | (5.66 – 28.5) | (3.11 – 37.0) | (14.6 – 22.7) | (15.6 – 41.0) | (6.88 – 48.7) |  |
| **TACE†** % | 91.0 | 86.1 | 85.8 | 46.7 | 86.9 | 0.92 (ns) |
| **(range)** | (0.52 - 94.4) | (0.53 - 95.3) | (9.59 - 94.0) | (1.48 - 91.9) | (0.83 - 94.5) |  |
| **Classical** % | 80.8 | 79.2 | 77.5 | 81.4 | 80.0 | 0.89 (ns) |
| **(range)** | (47.1 – 86.3) | (36.3 – 87.3) | (64.4 – 87.4) | (72.5 – 90.6) | (57.3 – 86.7) |  |
| **Intermediate** % | 6.50 | 7.35 | 9.22 | 4.21 | 6.90 | 0.26 (ns) |
| **(range)** | (2.37 – 18.8) | (2.52 – 17.1) | (5.65 – 14.5) | (3.35 – 10.4) | (4.63 – 15.6) |  |
| **Non-classical** % | 5.08 | 4.77 | 6.17 | 5.52 | 5.54 | 0.98 (ns) |
| **(range)** | (1.92 – 46.5) | (1.50 – 55.9) | (2.40 – 18.0) | (1.01 – 19.4) | (0.96 – 21.1) |  |
| **HERV H3** Classical (FMI) | 1.12 | 1.07 | 0.93 | 1.04 | 1.05 | 0.08 (ns) |
| **(range)** | (0.92 - 1.28) | (0.86 - 1.21) | (0.68 - 1.11) | (0.78 - 1.26) | (0.87 - 1.36) |  |
| **HERV H3** Intermediate (FMI) | 0.96 | 0.96 | 0.88 | 1.10 | 0.96 | 0.37 (ns) |
| **(range)** | (0.77 - 1.28) | (0.60 - 1.13) | (0.69 - 1.01) | (0.63 - 1.30) | (0.71 - 1.12) |  |
| **HERV H3** Non-classical (FMI) | 0.99* | 1.03* | 1.00* | 1.25 | 1.17* | 0.04 (*) |
| **(range)** | (0.81 - 1.26) | (0.81 - 1.62) | (0.62 - 1.36) | (0.79 - 2.04) | (0.77 - 2.29) |  |
| **HERV W3** Classical (FMI) | 0.99 | 0.98 | 1.00 | 0.95 | 0.94 | 0.73 (ns) |
| **(range)** | (0.56 - 1.27) | (0.75 - 1.11) | (0.81 - 1.09) | (0.83 - 1.11) | (0.77 - 1.44) |  |
| **HERV W3** Intermediate (FMI) | 0.94 | 0.92 | 0.99 | 0.94 | 0.91 | 0.37 (ns) |
| **(range)** | (0.60 - 1.31) | (0.69 - 1.14) | (0.76 - 1.11) | (0.68 - 1.52) | (0.80 - 1.43) |  |
| **HERV W3** Non-classical (FMI) | 1.00 | 1.04 | 1.19 | 1.17 | 1.13 | 0.31 (ns) |
| **(range)** | (0.72 - 1.64) | (0.76 - 1.87) | (0.71 - 1.86) | (0.86 - 2.24) | (0.87 - 1.90) |  |

**Supplementary table I3. The median levels and range of the total number of monocytes, the CD11b+, CD18+, CD40+, CD64+, CD86+, CD163+, CCR1+, CCR2+, CCR5+, and TACE+ monocytes; the three monocyte subsets: classical, intermediate, and non-classical; and the fluorescence median index of HERV H3 Env and HERV W3 Env expression on the three monocyte subsets.** Kruskal-Wallis with Dunn’s multiple comparisons test (alpha=0.05) was used to calculate significant differences, where (*) denotes significance in relation to the symptomatic control group. On average, fluorescent signals from more than 100.000 monocytes were collected for further analysis from each patient group (CIS, n=22; RRMS, n=33; PMS, n=6; RIS, n=6; SC, n=22).

**†** Results for TACE were only available for some of the participants (CIS, n=17; RRMS, n=25; PMS, n=6; RIS, n=2; SC, n=20). CIS (clinically isolated syndrome), RRMS (relapsing-remitting MS), PMS (progressive MS), RIS (radiologically isolated syndrome), SC (symptomatic controls), KW (Kruskal-Wallis), n (number of subjects), FMI (fluorescence median index = median fluorescence of immune sera / median fluorescence of appropriate control (pre-immune sera)).

| **Supplementary table I4.** **Variation in cell surface marker expression between patient groups and correlations to clinical disease measures** | | | | | | | | | | | | | | | | |
| --- | --- | --- | --- | --- | --- | --- | --- | --- | --- | --- | --- | --- | --- | --- | --- | --- |
| **Characteristics** | **CD11b** | **CD18** | **CD40** | **CD64** | **CD86** | **CD163** | **CCR1** | **CCR2** | **CCR5** | **TACE^†^** | **HERV H3**  (Cl.) | **HERV H3** (Int.) | **HERV**  **H3**  (Non-cl.) | **HERV W3** (Cl.) | **HERV W3** (Int.) | **HERV**  **W3** (Non-cl.) |
| ***P* values for Mann-Whitney’s *U*-test of differential expression of cell surface markers between MS relevant groups** | | | | | | | | | | | | | | | | |
| **RRMS vs PMS** (n=39) | 0.35 | 0.86 | 0.82 | 0.95 | 0.79 | 0.80 | 0.70 | 0.47 | 0.68 | 0.80 | 0.06 | 0.12 | 0.53 | 0.70 | 0.39 | 0.59 |
| **Level of significance** | - | - | - | - | - | - | - | - | - | - | - | - | - | - | - | - |
| **MS vs CIS** (n=61) | 0.56 | 0.76 | 0.06 | 0.71 | 0.76 | 0.12 | 0.41 | 0.98 | 0.20 | 0.55 | 0.01 | 0.27 | 0.38 | 0.91 | 0.86 | 0.41 |
| **Level of significance** | - | - | - | - | - | - | - | - | - | - | * | - | - | - | - | - |
| **RIS vs SC** (n=28) | 0.10 | 0.40 | 0.15 | 0.61 | 0.67 | 0.07 | 0.54 | 0.96 | 0.09 | 0.82 | 0.87 | 0.29 | >0.99 | 0.72 | 0.90 | 0.86 |
| **Level of significance** | - | - | - | - | - | - | - | - | - | - | - | - | - | - | - | - |
| **MS+CIS vs SC** (n=83) | 0.64 | 0.82 | 0.32 | 0.57 | 0.69 | 0.72 | 0.86 | 0.45 | 0.60 | 0.58 | 0.91 | 0.98 | <0.00 | 0.16 | 0.91 | 0.06 |
| **Level of significance** | - | - | - | - | - | - | - | - | - | - | - | - | ** | - | - | - |
| **MS+CIS vs SC+RIS** (n=89) | 0.24 | 0.60 | 0.55 | 0.43 | 0.88 | 0.70 | 0.66 | 0.37 | 0.76 | 0.52 | 0.81 | 0.69 | <0.00 | 0.19 | 0.99 | 0.06 |
| **Level of significance** | - | - | - | - | - | - | - | - | - | - | - | - | ** | - | - | - |
| **Gender**^a^ (n=61) | 0.35 | 0.25 | 0.14 | 0.56 | 0.80 | 0.29 | 0.79 | 0.27 | 0.65 | 0.83 | 0.20 | 0.11 | 0.62 | 0.92 | 0.43 | 0.18 |
| **Level of significance** | - | - | - | - | - | - | - | - | - | - | - | - | - | - | - | - |
| **Oligo clonal bands**^b^ (n=61) | 0.30 | 0.45 | 0.37 | 0.19 | 0.36 | 0.41 | 0.24 | 0.30 | 0.45 | 0.72 | 0.40 | 0.46 | 0.65 | 0.42 | 0.39 | 0.13 |
| **Level of significance** | - | - | - | - | - | - | - | - | - | - | - | - | - | - | - | - |
| **Progressed at followup**^c^ (n=61) | 0.38 | 0.43 | 0.85 | 0.14 | 0.62 | 0.44 | 0.44 | 0.50 | 0.60 | 0.40 | 0.92 | 0.40 | 0.78 | 0.18 | 0.15 | 0.61 |
| **Level of significance** | - | - | - | - | - | - | - | - | - | - | - | - | - | - | - | - |
| **Medication at followup**^d^ (n=61) | 0.27 | 0.60 | 0.36 | 0.57 | 0.80 | 0.79 | 0.70 | 0.39 | 0.22 | 0.82 | 0.42 | 0.32 | 0.18 | 0.96 | 0.96 | 0.78 |
| **Level of significance** | - | - | - | - | - | - | - | - | - | - | - | - | - | - | - | - |

**Supplementary table I4. Variation in cell surface marker expressions between patient groups and correlations to clinical disease measures.** First, each soluble marker value was compared for the five groups: RRMS (n=33) vs PMS (n=6); MS (RRMS + PMS, n=39) vs CIS (n=22); RIS (n=6) vs SC (n=22); MS + CIS (n=61) vs SC (n=22); and MS + CIS (n=61) vs SC + RIS (n=28). Then, each soluble marker was correlated to; age; gender, male (n=19) vs female (n=42); oligo clonal bands, yes (n=54) vs no (n=7); progressed, yes (n=26) vs no (n=35); and medication at followup, yes (n=41) vs no (n=20). The *P* values of the Mann-Whitneys *U*-test are shown with levels of significance: *<0.05, **<0.01, and *** <0.001. ^a,b^ Comparisons were only made for patients with CIS, RRMS and PMS. ^c^ Progressed vs non-progressed was estimated on the basis of a clinically defined attacks or a sustained increase of more than 0.5 in the EDSS scale within a follow-up period of 9 – 41 months (median=25). ^d^ Individuals who received immune-modulating therapy during the follow-up period versus individuals that did not. † Results for TACE were only available for 49 patients and 21 controls (CIS, n=16; RRMS, n=25; PMS, n=6; RIS, n=2; SC, n=21). CD11b, CD18, CD40, CD64, CD86, CD163, CCR1, CCR2, CCR5, and TACE positive monocytes; HERV H3 Env and HERV W3 Env expression on each of the three monocyte subsets (classical, intermediate, and non-classical monocytes). CIS (clinically isolated syndrome), RRMS (relapsing-remitting MS), PMS (progressive MS), RIS (radiologically isolated syndrome), SC (symptomatic controls), n (number of subjects).

**Supplementary figure I2**

**
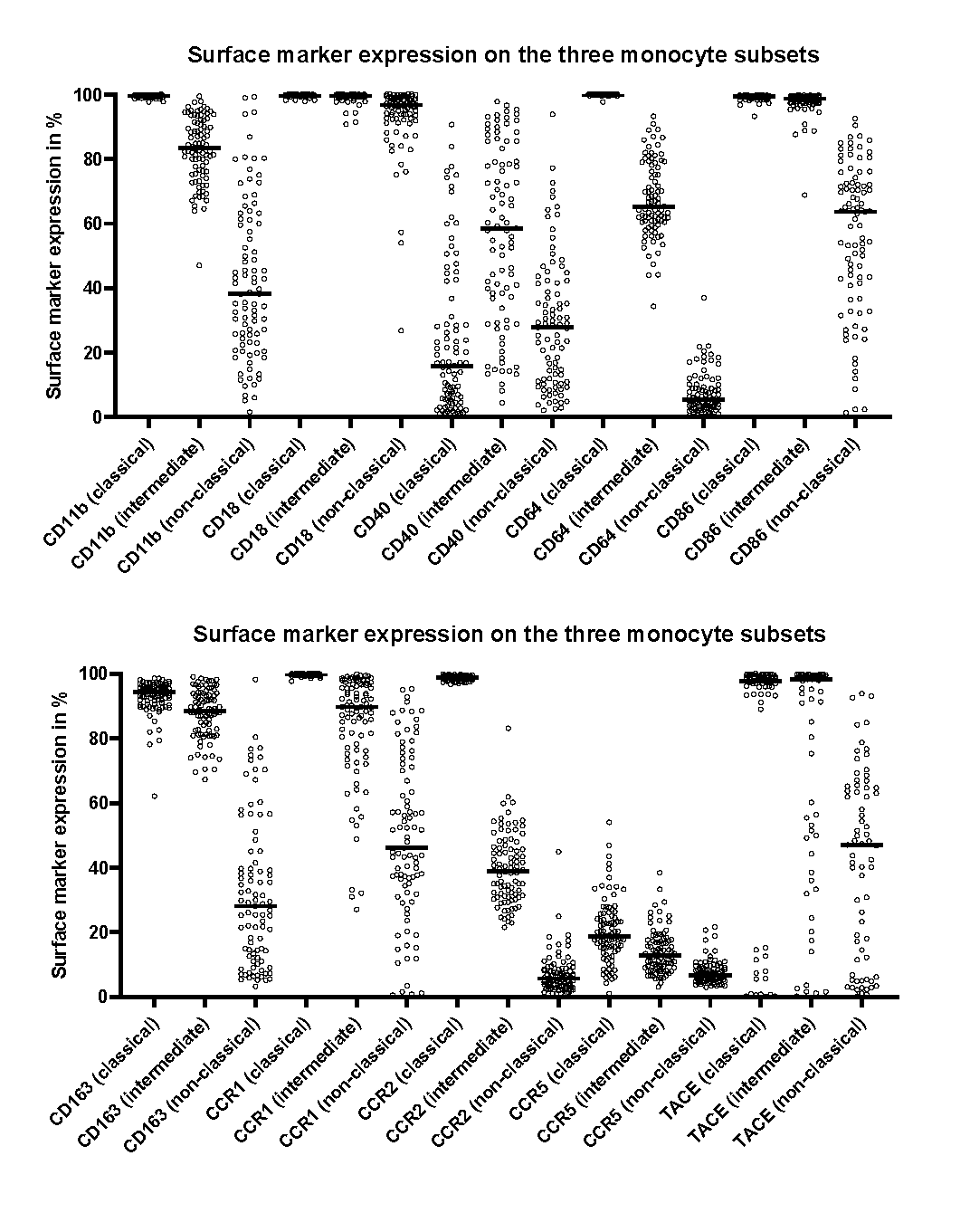
**

**Supplementary figure I2. Surface marker expression of CD11b, CD18, CD40, CD64, CD86, CD163, CCR1, CCR2, CCR5 and TACE on the three monocyte subsets.** The differences in expression of CD11b, CD18, CD40, CD64, CD86, CD163, CCR1, CCR2, CCR5 and TACE on the three monocyte subsets: classical, intermediate, and non-classical, were determined as % positive cells of the total monocytes from each patient. No significant differences were seen when comparing patients with MS + CIS and SC + RIS with respect to expression of the selected surface markers on the three monocyte subsets. Bars represent the median of the populations.

| **Supplementary table I5. Variation in soluble CSF markers of activation and inflammation between patient groups and clinical disease measures** | | | | | | | | | | | | | | | | | |
| --- | --- | --- | --- | --- | --- | --- | --- | --- | --- | --- | --- | --- | --- | --- | --- | --- | --- |
| **Characteristics** | **IFNγ** | **IL-1β** | **IL-2** | **IL-6** | **IL-8** | **IL-10** | **TNFα** | **GM-CSF** | **IL-5** | **IL-7** | **IL-12/ IL-23 p40** | **IL-15** | **IL-17A** | **MIP-1β** | **MCP-1** | **MCP-4** | **VEGF** |
| ***P* values for Mann-Whitney’s *U*-test of differential measures of soluble markers in cerebrospinal fluid** | | | | | | | | | | | | | | | | | |
| **RRMS vs PMS** (n=39) | 0.15 | 0.36 | 0.20 | 0.51 | 0.82 | 0.35 | 0.85 | 0.44 | 0.39 | 0.19 | 0.01 | 0.39 | 0.27 | 0.53 | 0.31 | 0.38 | 0.09 |
| **Level of significance** | - | - | - | - | - | - | - | - | - | - | * | - | - | - | - | - | - |
| **MS vs CIS** (n=61) | 0.95 | 0.93 | 0.07 | 0.69 | 0.67 | 0.75 | 0.73 | 0.11 | 0.78 | 0.96 | 0.89 | 0.11 | 0.68 | 0.31 | 0.59 | 0.36 | 0.07 |
| **Level of significance** | - | - | - | - | - | - | - | - | - | - | - | - | - | - | - | - | - |
| **RIS vs SC** (n=28) | 0.13 | 0.63 | 0.46 | 0.40 | 0.96 | 0.43 | 0.21 | 0.78 | 0.58 | 0.07 | 0.02 | 0.61 | 0.37 | 0.37 | 0.67 | 0.34 | 0.84 |
| **Level of significance** | - | - | - | - | - | - | - | - | - | - | * | - | - | - | - | - | - |
| **MS+CIS vs SC** (n=83) | <0.00 | <0.00 | 0.13 | 0.30 | <0.00 | <0.00 | <0.00 | 0.72 | 0.56 | <0.00 | <0.00 | 0.10 | <0.00 | <0.00 | 0.77 | 0.41 | <0.00 |
| **Level of significance** | *** | *** | - | - | *** | *** | *** | - | - | *** | *** | - | ** | *** | - | - | ** |
| **MS+CIS vs SC+RIS** (n=89) | <0.00 | <0.00 | 0.20 | 0.42 | <0.00 | <0.00 | <0.00 | 0.65 | 0.59 | <0.00 | <0.00 | 0.04 | <0.00 | <0.00 | 0.79 | 0.20 | <0.00 |
| **Level of significance** | *** | *** | - | - | *** | *** | *** | - | - | *** | *** | * | ** | *** | - | - | *** |
| **Gender**^a^ (n=61) | 0.17 | 0.87 | 0.76 | 0.08 | 0.15 | 0.73 | 0.53 | 0.22 | 0.08 | 0.72 | 0.37 | <0.00 | 0.80 | 0.47 | 0.10 | 0.28 | 0.63 |
| **Level of significance** | - | - | - | - | - | - | - | - | - | - | - | ** | - | - | - | - | - |
| **Oligo clonal bands**^b^ (n=61) | 0.23 | 0.12 | 0.74 | 0.82 | 0.05 | 0.06 | 0.15 | 0.15 | 0.53 | 0.06 | 0.01 | 0.77 | 0.85 | 0.51 | 0.73 | 0.54 | 0.26 |
| **Level of significance** | - | - | - | - | - | - | - | - | - | - | ** | - | - | - | - | - | - |
| **Progressed at followup**^c^ (n=61) | 0.33 | 0.13 | 0.43 | 0.23 | 0.28 | 0.99 | 0.71 | 0.03 | 0.96 | 0.98 | 0.86 | 0.68 | 0.68 | 0.27 | 0.02 | 0.11 | 0.86 |
| **Level of significance** | - | - | - | - | - | - | - | * | - | - | - | - | - | - | * | - | - |
| **Medication at followup**^d^ (n=61) | 0.18 | 0.52 | 0.04 | 0.64 | 0.08 | 0.09 | 0.19 | 0.49 | 0.89 | 0.13 | 0.01 | 0.07 | 0.98 | 0.34 | 0.95 | 0.44 | 0.78 |
| **Level of significance** | - | - | * | - | - | - | - | - | - | - | ** | - | - | - | - | - | - |

**Supplementary table I5. Variation in soluble CSF markers of activation and inflammation between patient groups and correlations to clinical disease measures.** First, each soluble marker value was compared for the five MS relevant groups: RRMS (n=33) vs PMS (n=6); MS (RRMS + PMS, n=39) vs CIS (n=22); RIS (n=6) vs SC (n=22); MS + CIS (n=61) vs SC (n=22); and MS + CIS (n=61) vs SC + RIS (n=28). Then, each soluble marker was correlated to; age; gender, male (n=19) vs female (n=42); oligo clonal bands, yes (n=54) vs no (n=7); progressed, yes (n=26) vs no (n=35); and medication at followup, yes (n=41) vs no (n=20). The *P* values of the Mann-Whitneys *U*-test are shown with levels of significance: *<0.05, **<0.01, and *** <0.001. ^a,b^ Comparisons were only made for patients with CIS, RRMS and PMS. ^c^ Progressed vs non-progressed was estimated on the basis of a clinically defined attacks or a sustained increase of more than 0.5 in the EDSS scale during a follow-up period of 9 – 41 months (median=25). ^d^ Individuals receiving immune-modulating therapy within the follow-up period versus individuals that did not. IFNγ (interferon gamma), IL-1β (interleukin-1β), IL-2 (interleukin-2), IL-6 (interleukin-6), IL-8 (interleukin-8), IL-10 (interleukin-10), TNFα (tumor necrosis factor alpha), GM-CSF (granulocyte-macrophage colony stimulating factor), IL-5 (interleukin-5), IL-7 (interleukin-7), IL-12/IL-23p40 (interleukin-12/interleukin 23 p40), IL-15 (interleukin-15), IL-17A (interleukin-17A), MIP-1β (macrophage inflammatory protein-1β), MCP-1 (monocyte chemoattractant protein-1), MCP-4 (monocyte chemoattractant protein-4), VEGF (vascular endothelial factor), CIS (clinically isolated syndrome), RRMS (relapsing-remitting MS), PMS (progressive MS), RIS (radiologically isolated syndrome), SC (symptomatic controls), n (number of subjects).

| **Supplementary table I6. Variation in soluble serum markers of activation and inflammation between relevant patient groups and clinical disease measures** | | | | | | | | | | | | | | | | | |
| --- | --- | --- | --- | --- | --- | --- | --- | --- | --- | --- | --- | --- | --- | --- | --- | --- | --- |
| **Characteristics** | **IFNγ** | **IL-1β** | **IL-2** | **IL-6** | **IL-8** | **IL-10** | **TNFα** | **GM-CSF** | **IL-5** | **IL-7** | **IL-12/IL-23p40** | **IL-15** | **IL-17A** | **MIP-1β** | **MCP1** | **MCP4** | **VEGF** |
| ***P* values for Mann-Whitney’s *U*-test of differential measures of soluble markers in serum** | | | | | | | | | | | | | | | | | |
| **RRMS vs PMS** (n=39) | 0.23 | 0.84 | 0.01 | 0.79 | 0.14 | 0.37 | 0.26 | 0.91 | 0.22 | 0.21 | 0.10 | 0.17 | 0.09 | 0.33 | 0.46 | 0.26 | 0.56 |
| **Level of significance** | - | - | ** | - | - | - | - | - | - | - | - | - | - | - | - | - | - |
| **MS vs CIS** (n=61) | 0.41 | 0.75 | 0.09 | 0.38 | 0.90 | 0.81 | 0.19 | 0.04 | 0.93 | 0.03 | 0.31 | 0.06 | 0.95 | 0.95 | 0.65 | 0.49 | 0.89 |
| **Level of significance** | - | - | - | - | - | - | - | * | - | * | - | - | - | - | - | - | - |
| **RIS vs SC** (n=28) | 0.76 | 0.61 | 0.80 | 0.10 | 0.24 | 0.23 | 0.37 | 0.25 | 0.43 | 0.02 | 0.02 | 0.78 | 0.04 | >0.99 | 0.07 | 0.02 | 0.37 |
| **Level of significance** | - | - | - | - | - | - | - | - | - | * | * | - | * | - | - | * | - |
| **MS+CIS vs SC** (n=83) | 0.34 | 0.25 | 0.72 | 0.73 | 0.29 | 0.91 | 0.62 | 0.14 | 0.17 | 0.19 | 0.24 | 0.44 | 0.88 | 0.42 | 0.12 | 0.20 | 0.89 |
| **Level of significance** | - | - | - | - | - | - | - | - | - | - | - | - | - | - | - | - | - |
| **MS+CIS vs SC+RIS** (n=89) | 0.35 | 0.16 | 0.79 | 0.83 | 0.64 | 0.57 | 0.81 | 0.30 | 0.08 | 0.02 | 0.84 | 0.34 | 0.40 | 0.37 | 0.30 | 0.70 | 0.60 |
| **Level of significance** | - | - | - | - | - | - | - | - | - | * | - | - | - | - | - | - | - |
| **Gender**^a^ (n=61) | 0.78 | 0.88 | 0.76 | 0.39 | 0.30 | 0.58 | 0.12 | 0.10 | 0.76 | 0.44 | 0.98 | 0.26 | 0.95 | 0.01 | 0.51 | 0.39 | 0.72 |
| **Level of significance** | - | - | - | - | - | - | - | - | - | - | - | - | - | * | - | - | - |
| **Oligo clonal bands**^b^ (n=61) | 0.19 | 0.30 | 0.66 | 0.68 | 0.03 | 0.33 | 0.34 | 0.17 | 0.87 | 0.21 | 0.89 | 0.77 | 0.91 | 0.18 | 0.80 | 0.39 | >0.99 |
| **Level of significance** | - | - | - | - | * | - | - | - | - | - | - | - | - | - | - | - | - |
| **Progressed at followup**^c^ (n=61) | 0.75 | 0.34 | 0.09 | 0.83 | 0.42 | 0.41 | 0.59 | 0.25 | 0.99 | 0.49 | 0.93 | 0.13 | 0.77 | 0.06 | 0.47 | 0.55 | 0.84 |
| **Level of significance** | - | - | - | - | - | - | - | - | - | - | - | - | - | - | - | - | - |
| **Medication at followup**^d^ (n=61) | 0.60 | 0.50 | <0.00 | 0.46 | 0.48 | 0.77 | 0.52 | 0.76 | 0.35 | 0.42 | 0.72 | 0.26 | 0.60 | 0.26 | 0.71 | 0.98 | 0.32 |
| **Level of significance** | - | - | ** | - | - | - | - | - | - | - | - | - | - | - | - | - | - |

**Supplementary table I6. Variation in soluble serum markers of activation and inflammation between MS relevant groups and correlations to clinical disease measures.** First, each soluble marker value was compared for the five MS relevant groups: RRMS (n=33) vs PMS (n=6); MS (RRMS + PMS, n=39) vs CIS (n=22); RIS (n=6) vs SC (n=22); MS + CIS (n=61) vs SC (n=22); and MS + CIS (n=61) vs SC + RIS (n=28). Then, each soluble marker was correlated to; age; gender, male (n=19) vs female (n=42); oligo clonal bands, yes (n=54) vs no (n=7); progressed, yes (n=26) vs no (n=35); and medication at followup, yes (n=41) vs no (n=20). The *P* values of the Mann-Whitneys *U*-test are shown with levels of significance: *<0.05, **<0.01, and *** <0.001. ^a,b,c,d^ Comparisons were only made for patients with CIS, RRMS and PMS. ^c^ Progressed vs non-progressed was estimated on the basis of a clinically defined attacks or a sustained increase of more than 0.5 in the EDSS scale within a follow-up period of 9 – 41 months (median=25). ^d^ Individuals receiving immune-modulating therapy within the follow-up period versus individuals that did not. IFNγ (interferon gamma), IL-1β (interleukin-1β), IL-2 (interleukin-2), IL-6 (interleukin-6), IL-8 (interleukin-8), IL-10 (interleukin-10), TNFα (tumor necrosis factor alpha), GM-CSF (granulocyte-macrophage colony stimulating factor), IL-5 (interleukin-5), IL-7 (interleukin-7), IL-12/IL-23p40 (interleukin-12/interleukin-23 p40), IL-15 (interleukin-15), IL-17A (interleukin-17A), MIP-1β (macrophage inflammatory protein-1β), MCP-1 (monocyte chemoattractant protein-1), MCP-4 (monocyte chemoattractant protein-4), VEGF (vascular endothelial factor), CIS (clinically isolated syndrome), RRMS (relapsing-remitting MS), PMS (progressive MS), RIS (radiologically isolated syndrome), SC (symptomatic controls), n (number of subjects).

| **Supplementary table I7. Median levels and range of soluble markers of activation and inflammation in serum, CSF, and their respective ratios as determined by MULTIPLEX analysis for the five patient groups.** | | | | | | |
| --- | --- | --- | --- | --- | --- | --- |
| **Characteristics** | **CIS** | **RRMS** | **PMS** | **RIS** | **SC** | **KW p-value**  **(significance)** |
| **No. of Subjects (total=90)** | n = 22 | n = 33 | n = 6 | n = 6 | n = 22 |  |
| **CSF IFNγ** (pg ml^-1^) | 0.29* | 0.32* | 0.00 | 0.04 | 0.00* | <0.00 (*) |
| **(range)** | (0.0 – 23) | (0.0 – 9.2) | (0.0 – 0.9) | (0.0 – 0.4) | (0.0 – 0.3) |  |
| **Serum IFNγ** (pg ml^-1^) | 3.80 | 2.60 | 2.50 | 3.00 | 2.43 | 0.51 (ns) |
| **(range)** | (0.9 - 53) | (1.3 - 21) | (1.2 - 3.6) | (1.1 - 9.6) | (0.9 - 73) |  |
| **IFNγ ratio** | 0.03* | 0.08* | 0.00 | 0.00 | 0.00* | 0.02 (*) |
| **(range)** | (0.0 - 11) | (0.0 – 2.6) | (0.0 - 0.3) | (0.0 - 0.2) | (0.0 – 0.1) |  |
| **CSF IL-1β** (pg ml^-1^) | 0.08* | 0.08* | 0.10* | 0.04 | 0.05* | <0.00 (*) |
| **(range)** | (0.0 - 0.3) | (0.0 - 2.6) | (0.1 - 0.2) | (0.0 - 0.2) | (0.0 - 0.2) |  |
| **Serum IL-1β** (pg ml^-1^) | 0.05 | 0.06 | 0.06 | 0.09 | 0.07 | 0.68 (ns) |
| **(range)** | (0.0 - 0.4) | (0.0 - 2.1) | (0.0 - 0.2) | (0.0 - 10.8) | (0.0 - 7.4) |  |
| **IL-1β ratio** | 1.57 | 1.57* | 2.04 | 0.56 | 1.03* | 0.04 (*) |
| **(range)** | (0.2 - 475) | (0.1 – 25554) | (0.5 – 795) | (0.0 - 1.6) | (0.0 – 939) |  |
| **CSF IL-2** (pg ml^-1^) | 0.15 | 0.29 | 0.12 | 0.19 | 0.18 | 0.11 (ns) |
| **(range)** | (0.0 - 0.6) | (0.0 - 0.5) | (0.0 - 0.7) | (0.0 - 16.5) | (0.0 - 0.4) |  |
| **Serum IL-2** (pg ml^-1^) | 0.00 | 0.04 | 0.0* | 0.0 | 0.0* | 0.05 (*) |
| **(range)** | (0.0 - 0.5) | (0.0 - 5.0) | (0.0 – 0.0) | (0.0 - 1.8) | (0.0 - 4.0) |  |
| **IL-2 ratio** | 393 | 3.26 | 1218 | 712 | 1.62 | 0.41 (ns) |
| **(range)** | (0.7 - 3392) | (0.0 - 3031) | (1 - 7400) | (0.0 - 165454) | (0.0 - 3818) |  |
| **CSF IL-6** (pg ml^-1^) | 1.24 | 1.14 | 1.42 | 1.31 | 1.16 | 0.76 (ns) |
| **(range)** | (0.3 - 4.3) | (0.5 - 3.8) | (0.7 - 1.6) | (0.8 - 1.9) | (0.5 - 1.9) |  |
| **Serum IL-6** (pg ml^-1^) | 0.86 | 0.78 | 0.85 | 1.12 | 0.74 | 0.55 (ns) |
| **(range)** | (0.2 - 5.3) | (0.3 - 5.9) | (0.4 - 2.5) | (0.6 - 23) | (0.2 - 5.5) |  |
| **IL-6 ratio** | 1.45 | 1.61 | 1.67 | 1.09 | 1.50 | 0.78 (ns) |
| **(range)** | (0.1 - 13) | (0.2 – 8.2) | (0.6 - 3.0) | (0.1 - 3.1) | (0.1 – 5.6) |  |
| **CSF IL-8** (pg ml^-1^) | 41.9* | 41.6* | 36.9* | 28.3 | 28.8* | <0.00 (*) |
| **(range)** | (22 – 162) | (23 - 64) | (27 - 80) | (23 - 51) | (20 - 43) |  |
| **Serum IL-8** (pg ml^-1^) | 10.4 | 9.70 | 13.0 | 12.0 | 9.63 | 0.36 (ns) |
| **(range)** | (4.9 - 23) | (5.7 - 217) | (9.0 - 15) | (3.1- 1578) | (4.4 - 800) |  |
| **IL-8 ratio** | 4.37* | 4.20* | 3.05 | 2.37 | 3.33* | 0.04 (*) |
| **(range)** | (1.5 - 10) | (0.2 – 7.7) | (2.3 - 8.9) | (0.0 - 16) | (0.0 – 5.5) |  |
| **CSF IL-10** (pg ml^-1^) | 0.19* | 0.18* | 0.15* | 0.10 | 0.07* | <0.00 (*) |
| **(range)** | (0.0 - 0.9) | (0.1 - 0.6) | (0.1 - 0.3) | (0.0 - 0.2) | (0.0 - 0.2) |  |
| **Serum IL-10** (pg ml^-1^) | 0.40 | 0.41 | 0.36 | 0.77 | 0.38 | 0.67 (ns) |
| **(range)** | (0.2 - 1.8) | (0.2 - 3.7) | (0.2 - 0.4) | (0.2 - 2.6) | (0.2 - 7.9) |  |
| **IL-10 ratio** | 0.41* | 0.46* | 0.47* | 0.15 | 0.23* | <0.00 (*) |
| **(range)** | (0.1 – 2.8) | (0.1 – 1.7) | (0.2 - 1.1) | (0.0 - 0.6) | (0.0 – 0.7) |  |
| **CSF TNFα** (pg ml^-1^) | 0.26* | 0.26* | 0.27* | 0.20 | 0.16* | <0.00 (*) |
| **(range)** | (0.1 - 0.7) | (0.2 - 0.6) | (0.2 - 0.4) | (0.2 - 0.3) | (0.1 - 0.3) |  |
| **Serum TNFα** (pg ml^-1^) | 2.30 | 2.46 | 2.35 | 2.45 | 2.39 | 0.55 (ns) |
| **(range)** | (0.5 - 4.2) | (1.7 - 3.7) | (1.9 - 2.6) | (2.1 - 12) | (1.1 - 3.7) |  |
| **TNFα ratio** | 0.12* | 0.11* | 0.12* | 0.08 | 0.07* | <0.00 (*) |
| **(range)** | (0.1 – 0.3) | (0.0 – 0.3) | (0.1 - 0.2) | (0.0 - 0.1) | (0.0 – 0.2) |  |
| **CSF GM-CSF** (pg ml^-1^) | 0.10 | 0.07 | 0.10 | 0.10 | 0.09 | 0.49 (ns) |
| **(range)** | (0.0 - 0.3) | (0.0 - 0.3) | (0.0 - 0.2) | (0.0 - 0.2) | (0.0 - 0.2) |  |
| **Serum GM-CSF** (pg ml^-1^) | 0.14 | 0.11 | 0.10 | 0.07 | 0.17 | 0.16 (ns) |
| **(range)** | (0.0 - 0.3) | (0.0 - 0.3) | (0.0 - 0.2) | (0.0 - 0.7) | (0.0 - 0.4) |  |
| **GM-CSF ratio** | 0.76 | 0.89 | 0.85 | 1.27 | 0.74 | 0.87 (ns) |
| **(range)** | (0.0 – 2527) | (0.0 - 1861) | (0.0 - 38) | (0.2 - 2.9) | (0.0 - 425) |  |
| **CSF IL-5** (pg ml^-1^) | 0.41 | 0.47 | 0.36 | 0.41 | 0.48 | 0.83 (ns) |
| **(range)** | (0.2 - 0.7) | (0.2 - 18) | (0.2 - 0.8) | (0.2 - 0.8) | (0.2 - 0.7) |  |
| **Serum IL-5** (pg ml^-1^) | 0.12 | 0.11 | 0.24 | 0.23 | 0.17 | 0.27 (ns) |
| **(range)** | (0.0 - 1.7) | (0.0 - 0.4) | (0.0 - 0.4) | (0.0 - 0.4) | (0.0 - 1.9) |  |
| **IL-5 ratio** | 3.46 | 4.45 | 1.94 | 1.86 | 2.90 | 0.27 (ns) |
| **(range)** | (0.3 - 3451) | (1.2 - 5375) | (1.0 - 2470) | (0.9 - 20) | (0.3 - 6574) |  |
| **CSF IL-7** (pg ml^-1^) | 0.47* | 0.49* | 0.74 | 0.65 | 0.91* | <0.00 (*) |
| **(range)** | (0.1 - 1.6) | (0.1 - 1.1) | (0.4 - 1.2) | (0.5 - 1.1) | (0.4 - 1.5) |  |
| **Serum IL-7** (pg ml^-1^) | 12.8 | 15.1* | 20.9* | 7.98* | 13.1* | <0.00 (*) |
| **(range)** | (5.4 - 20) | (5.0 - 24) | (11 - 37) | (3.7 - 10) | (4.0 - 25) |  |
| **IL-7 ratio** | 0.05* | 0.03* | 0.04* | 0.07 | 0.07* | <0.00 (*) |
| **(range)** | (0.0 – 0.1) | (0.0 – 0.1) | (0.0 - 0.1) | (0.0 - 0.3) | (0.0 – 0.3) |  |
| **CSF IL-12/IL-23p40** (pg ml^-1^) | 9.47* | 13.3* | 4.38 | 1.99 | 3.91* | <0.00 (*) |
| **(range)** | (3.4 - 123) | (3.9 - 74) | (2.6 - 18) | (1.6 - 5.8) | (2.4 - 5.7) |  |
| **Serum IL-12/IL-23p40** (pg ml^-1^) | 82.9 | 112 | 79.9* | 55.1* | 97.7* | 0.04 (*) |
| **(range)** | (39 - 156) | (40 - 322) | (35 - 97) | (28 - 123) | (43 - 290) |  |
| **IL-12/IL-23p40 ratio** | 0.15* | 0.14* | 0.07* | 0.04 | 0.03* | <0.00 (*) |
| **(range)** | (0.0 – 0.9) | (0.0 – 0.6) | (0.0 - 0.2) | (0.0 - 0.1) | (0.0 – 0.1) |  |
| **CSF IL-15** (pg ml^-1^) | 2.43 | 2.77 | 2.34 | 2.21 | 2.32 | 0.08 (ns) |
| **(range)** | (1.6 - 3.9) | (1.6 - 4.4) | (1.4 - 5.0) | (1.9 - 2.8) | (1.4 - 4.1) |  |
| **Serum IL-15** (pg ml^-1^) | 2.24 | 2.50 | 2.87 | 2.18 | 2.18 | 0.20 (ns) |
| **(range)** | (1.4 - 6.2) | (1.3 - 3.9) | (2.0 - 3.4) | (1.3 – 2.9) | (1.1 - 4.5) |  |
| **IL-15 ratio** | 1.04 | 1.15 | 0.89 | 0.95 | 1.03 | 0.30 (ns) |
| **(range)** | (0.4 – 1.9) | (0.6 – 1.8) | (0.5 - 2.2) | (0.8 - 1.6) | (0.5 – 1.4) |  |
| **CSF IL-17A** (pg ml^-1^) | 0.26* | 0.22* | 0.17 | 0.18 | 0.14* | 0.04 (*) |
| **(range)** | (0.0 - 0.7) | (0.0 - 1.3) | (0.1 - 0.2) | (0.0 - 0.3) | (0.0 - 0.3) |  |
| **Serum IL-17A** (pg ml^-1^) | 1.28 | 1.33 | 0.96 | 0.82 | 1.39 | 0.08 (ns) |
| **(range)** | (0.6 - 11) | (0.5 - 5.2) | (0.8 - 1.4) | (0.6 - 1.1) | (0.4 - 6.8) |  |
| **IL-17A ratio** | 0.17* | 0.19* | 0.14 | 0.24* | 0.10* | 0.05 (*) |
| **(range)** | (0.0 – 0.5) | (0.0 – 1.0) | (0.1 - 0.2) | (0.0 - 0.3) | (0.0 – 0.3) |  |
| **CSF MIP-1β** (pg ml^-1^) | 10.9* | 12.1* | 12.8* | 8.33 | 9.12* | <0.00 (*) |
| **(range)** | (4.6 - 34) | (0.0 - 25) | (8.7 - 31) | (5.7 - 9.3) | (5.5 - 16) |  |
| **Serum MIP-1β** (pg ml^-1^) | 111 | 105 | 85.1 | 90.3 | 104 | 0.81 (ns) |
| **(range)** | (56 - 296) | (46 - 322) | (51 - 236) | (73 - 736) | (39 - 203) |  |
| **MIP-1β ratio** | 0.10* | 0.11* | 0.14* | 0.09 | 0.09* | 0.01 (*) |
| **(range)** | (0.1 – 0.2) | (0.0 – 0.3) | (0.1 - 0.2) | (0.0 - 0.1) | (0.0 – 0.2) |  |
| **CSF MCP-1** (pg ml^-1^) | 306 | 311 | 353 | 342 | 321 | 0.82 (ns) |
| **(range)** | (195 - 793) | (0.0 - 474) | (276 - 462) | (252 - 379) | (247 - 624) |  |
| **Serum MCP-1** (pg ml^-1^) | 240 | 244 | 298 | 261 | 225 | 0.31 (ns) |
| **(range)** | (107 - 515) | (150 - 609) | (146 - 324) | (197 - 482) | (119 - 598) |  |
| **MCP-1 ratio** | 1.29 | 1.29 | 1.29 | 1.33 | 1.48 | 0.51 (ns) |
| **(range)** | (0.7 – 3.6) | (0.0 – 2.5) | (1.0 - 2.0) | (0.6 - 1.5) | (0.5 – 2.5) |  |
| **CSF MCP-4** (pg ml^-1^) | 1.73 | 1.41 | 1.64 | 2.20 | 1.83 | 0.43 (ns) |
| **(range)** | (0.0 - 7.3) | (0.0 - 18) | (0.0 - 5.5) | (1.0 - 6.6) | (0.0 - 3.8) |  |
| **Serum MCP-4** (pg ml^-1^) | 119 | 104 | 145 | 150 | 101 | 0.10 (ns) |
| **(range)** | (43 - 259) | (42 - 294) | (60 - 215) | (105 - 328) | (31 - 239) |  |
| **MCP-4 ratio** | 0.02 | 0.01 | 0.01 | 0.01 | 0.01 | 0.83 (ns) |
| **(range)** | (0.0 – 0.1) | (0.0 – 0.2) | (0.0 - 0.0) | (0.0 - 0.0) | (0.0 – 0.1) |  |
| **CSF VEGF** (pg ml^-1^) | 2.47 | 2.53* | 3.05* | 1.90 | 2.24* | <0.00 (*) |
| **(range)** | (1.5 - 3.7) | (1.8 - 4.1) | (2.5 - 4.5) | (1.5 - 3.2) | (1.4 - 2.9) |  |
| **Serum VEGF** (pg ml^-1^) | 101 | 115 | 79.0 | 88.1 | 103 | 0.80 (ns) |
| **(range)** | (36 - 272) | (30 - 680) | (53 - 234) | (23 - 109) | (1.2 - 358) |  |
| **VEGF ratio** | 0.02 | 0.02 | 0.04 | 0.02 | 0.02 | 0.54 (ns) |
| **(range)** | (0.0 – 0.1) | (0.0 – 0.1) | (0.0 - 0.1) | (0.0 - 0.1) | (0.0 – 2.4) |  |

**Supplementary table I7. The median levels and range of soluble markers of activation and inflammation in serum and CSF, and their respective ratios.** Kruskal-Wallis with Dunn’s multiple comparisons test (alpha=0.05) was used to calculate significant differences, where (*) denotes significance in relation to the symptomatic control group. For statistical purposes, values below lower limit of detection (LLOD) were all substituted with 0.0001. CIS (clinically isolated syndrome), RRMS (relapsing-remitting MS), PMS (progressive MS), SC (symptomatic controls), KW (Kruskal-Wallis), n (number of subjects), IFNγ (interferon gamma), IL-1β (interleukin-1β), IL-2 (interleukin-2), IL-6 (interleukin-6), IL-8 (interleukin-8), IL-10 (interleukin-10), TNFα (tumor necrosis factor alpha), GM-CSF (granulocyte-macrophage colony stimulating factor), IL-5 (interleukin-5), IL-7 (interleukin-7), IL-12/IL-23p40 (interleukin-12/interleukin-23p40), IL-15 (interleukin-15), IL-17A (interleukin-17A), MIP-1β (macrophage inflammatory protein-1β), MCP-1 (monocyte chemoattractant protein-1), MCP-4 (monocyte chemoattractant protein-4), VEGF (vascular endothelial growth factor).

| **Supplementary table I8. Correlation between cell surface marker expression and soluble markers of activation and inflammation in serum and CSF as well as their respective ratios** | | | | | | | | | | | | | | | | |
| --- | --- | --- | --- | --- | --- | --- | --- | --- | --- | --- | --- | --- | --- | --- | --- | --- |
| **Characteristics** | **CD11b** | **CD18** | **CD40** | **CD64** | **CD86** | **CD163** | **CCR1** | **CCR2** | **CCR5** | **TACE** | **HERV H3**  (Cl.) | **HERV H3** (Int.) | **HERV**  **H3**  (Non-cl.) | **HERV W3** (Cl.) | **HERV W3** (Int.) | **HERV**  **W3** (Non-cl.) |
| **Spearman’s correlation coefficients, Rho, and level of significance *P*** | | | | | | | | | | | | | | | | |
| **CSF IFNγ** | 0,14207 | -0,05256 | 0,014451 | 0,208199 | 0,090593 | 0,12379 | 0,034836 | 0,154265 | 0,079376 | 0,051712 | 0,163861 | 0,089696 | -0,10596 | 0,120312 | -0,1392 | -0,04401 |
| **Level of significance** | 0,184151 | 0,624687 | 0,89308 | 0,050243 | 0,398499 | 0,247773 | 0,745865 | 0,148903 | 0,459657 | 0,670729 | 0,124931 | 0,403202 | 0,323018 | 0,269848 | 0,20116 | 0,687455 |
| **Serum IFNγ** | 0,028307 | -0,07904 | 0,133735 | 0,068219 | 0,035398 | -0,07303 | -0,09579 | -0,0858 | 0,224419 | -0,00052 | -0,10441 | -0,03112 | -0,15304 | 0,073278 | 0,079353 | 0,059824 |
| **Level of significance** | 0,792302 | 0,461556 | 0,211506 | 0,525283 | 0,741905 | 0,496406 | 0,371864 | 0,424011 | 0,034491 | 0,996559 | 0,330186 | 0,772191 | 0,152198 | 0,502534 | 0,467674 | 0,58427 |
| **IFNγ ratio** | 0,120642 | -0,04387 | -0,00259 | 0,184201 | 0,07402 | 0,122581 | 0,063544 | 0,172803 | 0,017179 | 0,03657 | 0,164012 | 0,052563 | -0,07555 | 0,087628 | -0,18175 | -0,089 |
| **Level of significance** | 0,260097 | 0,683127 | 0,980799 | 0,083987 | 0,490587 | 0,252459 | 0,554121 | 0,105365 | 0,873052 | 0,763749 | 0,12458 | 0,624698 | 0,481668 | 0,422392 | 0,093965 | 0,415156 |
| **CSF IL-1β** | 0,055355 | -0,14235 | -0,10253 | 0,13127 | 0,028588 | 0,023549 | 0,154696 | 0,054203 | -0,04638 | 0,198971 | 0,083948 | 0,04646 | 0,063153 | 0,118241 | 0,012161 | 0,047938 |
| **Level of significance** | 0,606395 | 0,183283 | 0,339009 | 0,220121 | 0,790288 | 0,826613 | 0,147758 | 0,613917 | 0,666021 | 0,098679 | 0,434124 | 0,665492 | 0,556568 | 0,27824 | 0,911511 | 0,661166 |
| **Serum IL-1β** | 0,079813 | -0,19749 | 0,048483 | -0,09728 | -0,17045 | -0,05343 | -0,08971 | -0,14952 | 0,150761 | 0,06352 | -0,15888 | -0,08897 | -0,10637 | -0,03551 | -0,04337 | -0,03552 |
| **Level of significance** | 0,457181 | 0,063583 | 0,651854 | 0,364475 | 0,110271 | 0,619005 | 0,403111 | 0,161964 | 0,158467 | 0,601389 | 0,136979 | 0,407053 | 0,321143 | 0,745477 | 0,691718 | 0,745445 |
| **IL-1β ratio** | -0,07323 | 0,100255 | -0,01677 | 0,103001 | 0,158489 | 0,002673 | 0,109578 | 0,08565 | -0,16137 | 0,038574 | 0,205239 | 0,12548 | 0,139823 | 0,132829 | 0,090637 | 0,033266 |
| **Level of significance** | 0,495224 | 0,349895 | 0,876053 | 0,336787 | 0,137957 | 0,980164 | 0,306676 | 0,424834 | 0,130852 | 0,751211 | 0,053676 | 0,241326 | 0,191258 | 0,222772 | 0,40657 | 0,761076 |
| **CSF IL-2** | 0,022125 | -0,04043 | 0,046206 | -0,04238 | -0,18665 | -0,11023 | -0,11121 | -0,04972 | 0,049021 | -0,09473 | 0,004992 | 0,038269 | 0,114234 | -0,10637 | -0,16551 | 0,054282 |
| **Level of significance** | 0,836946 | 0,70676 | 0,667214 | 0,693325 | 0,079886 | 0,303771 | 0,299491 | 0,643547 | 0,648245 | 0,435366 | 0,962968 | 0,721797 | 0,286449 | 0,329692 | 0,127783 | 0,61962 |
| **Serum IL-2** | 0,082608 | 0,026988 | 0,180356 | -0,04503 | -0,06011 | -0,08436 | -0,11918 | -0,00966 | 0,09278 | -0,11511 | -0,08275 | 0,00023 | 0,063777 | -0,09289 | -0,12876 | 0,101762 |
| **Level of significance** | 0,441522 | 0,801772 | 0,090776 | 0,675192 | 0,575751 | 0,431865 | 0,265972 | 0,928428 | 0,387158 | 0,342686 | 0,440726 | 0,998294 | 0,552668 | 0,394942 | 0,237386 | 0,351171 |
| **IL-2 ratio** | -0,10884 | 0,002102 | -0,09897 | 0,051381 | -0,02802 | 0,008513 | 0,046854 | -0,02186 | -0,02335 | -0,0231 | -0,05669 | -0,04717 | 0,015546 | -0,03468 | -0,00291 | -0,07659 |
| **Level of significance** | 0,309983 | 0,984401 | 0,35616 | 0,632513 | 0,794379 | 0,936894 | 0,662831 | 0,838866 | 0,828021 | 0,84945 | 0,597698 | 0,660715 | 0,885031 | 0,751251 | 0,97876 | 0,483336 |
| **CSF IL-6** | -0,21756 | 0,123814 | 0,106385 | -0,03829 | 0,04774 | 0,127157 | -0,07093 | -0,06016 | 0,043508 | 0,062966 | 0,120753 | 0,128364 | -0,00995 | 0,172699 | 0,105732 | -0,01893 |
| **Level of significance** | 0,040554 | 0,247682 | 0,321066 | 0,721669 | 0,656849 | 0,235044 | 0,508944 | 0,575479 | 0,68559 | 0,604571 | 0,259653 | 0,230594 | 0,926261 | 0,111816 | 0,332598 | 0,862685 |
| **Serum IL-6** | 0,210917 | -0,1826 | 0,032841 | 0,023892 | -0,04798 | -0,03645 | -0,12147 | -0,02616 | 0,182673 | 0,047963 | 0,050589 | 0,038586 | 0,099219 | 0,084681 | 0,18506 | 0,196685 |
| **Level of significance** | 0,047252 | 0,086762 | 0,75997 | 0,824122 | 0,655267 | 0,734542 | 0,256809 | 0,807702 | 0,086635 | 0,693367 | 0,637779 | 0,719587 | 0,354921 | 0,438221 | 0,088039 | 0,069514 |
| **IL-6 ratio** | -0,22451 | 0,174885 | 0,038602 | 0,023309 | 0,101031 | 0,163338 | 0,078084 | 0,030561 | -0,10098 | 0,005074 | 0,009108 | 0,010742 | -0,08327 | 0,055795 | -0,0772 | -0,15454 |
| **Level of significance** | 0,034415 | 0,101172 | 0,719475 | 0,828353 | 0,346159 | 0,126157 | 0,467014 | 0,776178 | 0,34642 | 0,966745 | 0,932491 | 0,920413 | 0,437887 | 0,609876 | 0,479866 | 0,155407 |
| **CSF IL-8** | 0,106892 | 0,02776 | -0,07493 | 0,108098 | 0,101542 | -0,04809 | 0,069546 | -0,0178 | -0,02321 | 0,191284 | 0,1857 | 0,140578 | -0,07053 | 0,115346 | 0,013821 | -0,14457 |
| **Level of significance** | 0,318753 | 0,796225 | 0,485241 | 0,313291 | 0,343714 | 0,654481 | 0,517243 | 0,868497 | 0,829102 | 0,112678 | 0,081454 | 0,188848 | 0,511308 | 0,290259 | 0,899491 | 0,184148 |
| **Serum IL-8** | -0,1035 | 0,058749 | 0,043608 | -0,11415 | -0,04435 | -0,01984 | -0,14144 | -0,11672 | 0,121407 | -0,13879 | -0,25058 | -0,14179 | -0,07123 | -0,1531 | -0,0752 | 0,078947 |
| **Level of significance** | 0,334421 | 0,584462 | 0,684911 | 0,286802 | 0,679829 | 0,853616 | 0,18612 | 0,276025 | 0,257063 | 0,251853 | 0,017862 | 0,185036 | 0,507127 | 0,159324 | 0,491351 | 0,46996 |
| **IL-8 ratio** | 0,180677 | -0,00164 | -0,10735 | 0,185533 | 0,125467 | 0,0201 | 0,161984 | 0,08695 | -0,06104 | 0,263655 | 0,321808 | 0,220481 | 0,020667 | 0,156282 | 0,021048 | -0,13772 |
| **Level of significance** | 0,090193 | 0,98786 | 0,316689 | 0,081733 | 0,241374 | 0,85169 | 0,129372 | 0,41781 | 0,569846 | 0,027429 | 0,002103 | 0,037872 | 0,847555 | 0,150736 | 0,847462 | 0,206034 |
| **CSF IL-10** | 0,047002 | 0,035514 | 0,071468 | 0,049091 | 0,112663 | 0,013119 | 0,078519 | -0,03562 | -0,05952 | 0,216695 | 0,238034 | 0,224416 | -0,07726 | 0,070627 | 0,008331 | -0,06638 |
| **Level of significance** | 0,661829 | 0,741092 | 0,505697 | 0,647779 | 0,293175 | 0,902881 | 0,46453 | 0,740331 | 0,579557 | 0,071572 | 0,024688 | 0,034494 | 0,471766 | 0,518156 | 0,939319 | 0,543679 |
| **Serum IL-10** | 0,050612 | -0,05394 | 0,068336 | 0,06078 | 0,140854 | 0,115511 | 0,132172 | 0,045417 | 0,186744 | 0,111166 | -0,02081 | 0,050495 | -0,13035 | -0,05732 | -0,11689 | -0,15678 |
| **Level of significance** | 0,637624 | 0,615665 | 0,524572 | 0,571522 | 0,18797 | 0,281061 | 0,216941 | 0,672572 | 0,079725 | 0,359575 | 0,846498 | 0,638405 | 0,223409 | 0,600101 | 0,283791 | 0,149414 |
| **IL-10 ratio** | 0,010769 | 0,022111 | 0,020335 | 0,015826 | 0,000255 | -0,0841 | -0,00499 | -0,07356 | -0,10647 | 0,137672 | 0,212343 | 0,114854 | -0,03029 | 0,087533 | 0,044181 | 0,038983 |
| **Level of significance** | 0,920214 | 0,837049 | 0,849974 | 0,882976 | 0,998105 | 0,433317 | 0,962992 | 0,493295 | 0,320691 | 0,255737 | 0,045743 | 0,283825 | 0,77814 | 0,422896 | 0,68627 | 0,721571 |
| **CSF TNFα** | 0,110291 | -0,00238 | -0,07137 | 0,123195 | 0,062712 | -0,00372 | 0,133024 | 0,055592 | -0,05156 | 0,254327 | 0,177861 | 0,147217 | -0,01413 | 0,189503 | 0,026522 | 0,008869 |
| **Level of significance** | 0,303519 | 0,982359 | 0,506262 | 0,250072 | 0,559334 | 0,972427 | 0,213964 | 0,604849 | 0,631302 | 0,03362 | 0,095406 | 0,168598 | 0,895433 | 0,080545 | 0,808473 | 0,935406 |
| **Serum TNFα** | 0,103504 | -0,1527 | 0,051916 | -0,08589 | -0,15654 | -0,12806 | -0,12125 | -0,21531 | 0,090847 | -0,02244 | -0,06602 | 0,009661 | -0,0422 | -0,0519 | 0,024086 | -0,10644 |
| **Level of significance** | 0,334419 | 0,153114 | 0,628972 | 0,42354 | 0,142928 | 0,231697 | 0,257676 | 0,042731 | 0,397169 | 0,853702 | 0,538754 | 0,9284 | 0,694548 | 0,635093 | 0,825771 | 0,329355 |
| **TNFα ratio** | 0,032614 | 0,07706 | -0,10266 | 0,144372 | 0,139116 | 0,068848 | 0,177367 | 0,142897 | -0,04272 | 0,252019 | 0,185087 | 0,136041 | 0,010163 | 0,124884 | -0,07033 | 0,03503 |
| **Level of significance** | 0,761575 | 0,472896 | 0,338376 | 0,177068 | 0,193534 | 0,521465 | 0,096345 | 0,181584 | 0,691021 | 0,03532 | 0,082483 | 0,203664 | 0,924689 | 0,251932 | 0,5199 | 0,748816 |
| **CSF GM-CSF** | -0,09294 | 0,049642 | -0,12687 | 0,035181 | 0,043851 | -0,06892 | 0,000145 | 0,074758 | 0,065919 | -0,0428 | 0,07537 | -0,05236 | -0,08517 | 0,040857 | -0,05938 | -0,13207 |
| **Level of significance** | 0,386352 | 0,644089 | 0,236127 | 0,743435 | 0,683246 | 0,521032 | 0,998924 | 0,486257 | 0,539377 | 0,724958 | 0,482686 | 0,626029 | 0,427436 | 0,708772 | 0,587078 | 0,225463 |
| **Serum GM-CSF** | -0,04356 | -0,07322 | -0,02961 | -0,1159 | -0,12254 | -0,24228 | -0,22579 | -0,07293 | 0,057558 | -0,18895 | -0,16024 | -0,10981 | -0,05744 | -0,14458 | -0,08599 | -0,04653 |
| **Level of significance** | 0,685227 | 0,495302 | 0,782984 | 0,279448 | 0,252614 | 0,022161 | 0,033378 | 0,497004 | 0,592118 | 0,117227 | 0,133603 | 0,305648 | 0,592911 | 0,18414 | 0,431139 | 0,670512 |
| **GM-CSF ratio** | -0,02901 | 0,151478 | -0,07135 | 0,095565 | 0,11355 | 0,120906 | 0,205879 | 0,083918 | 0,035435 | 0,084288 | 0,224682 | 0,085804 | 0,052189 | 0,074034 | -0,01669 | -0,02946 |
| **Level of significance** | 0,787295 | 0,156473 | 0,506402 | 0,373005 | 0,289364 | 0,259047 | 0,052917 | 0,434294 | 0,741651 | 0,487842 | 0,034274 | 0,423995 | 0,627165 | 0,498121 | 0,878775 | 0,787762 |
| **CSF IL-5** | 0,0876 | 0,11292 | -0,15031 | 0,070017 | 0,044163 | 0,0106 | 0,003733 | -0,03248 | -0,0181 | -0,00787 | 0,052762 | 0,069899 | 0,090159 | -0,01148 | 0,003868 | -0,01194 |
| **Level of significance** | 0,414326 | 0,292068 | 0,159729 | 0,514401 | 0,681113 | 0,921464 | 0,972301 | 0,762503 | 0,866278 | 0,948471 | 0,623382 | 0,515112 | 0,400771 | 0,916431 | 0,971801 | 0,913079 |
| **Serum IL-5** | 0,287943 | 0,140319 | 0,106132 | 0,250532 | 0,264169 | 0,256185 | 0,297481 | 0,149339 | -0,01994 | 0,168887 | -0,00284 | 0,148185 | 0,200996 | -0,0415 | 0,105068 | 0,114785 |
| **Level of significance** | 0,006214 | 0,18967 | 0,322229 | 0,017884 | 0,012365 | 0,015382 | 0,004638 | 0,162478 | 0,852859 | 0,162225 | 0,97896 | 0,165785 | 0,05893 | 0,704396 | 0,335663 | 0,292625 |
| **IL-5 ratio** | -0,2378 | -0,08654 | -0,14837 | -0,20673 | -0,22148 | -0,25367 | -0,29444 | -0,1557 | 0,062675 | -0,15708 | 0,012036 | -0,13803 | -0,20065 | 0,057182 | -0,09229 | -0,13333 |
| **Level of significance** | 0,024833 | 0,420043 | 0,16526 | 0,051927 | 0,036989 | 0,016457 | 0,005097 | 0,145124 | 0,559566 | 0,194077 | 0,910865 | 0,197058 | 0,05938 | 0,601004 | 0,398042 | 0,221022 |
| **CSF IL-7** | -0,14667 | -0,03186 | -0,00478 | -0,08675 | 0,061603 | 0,011323 | -0,01621 | -0,08765 | -0,02344 | -0,16737 | -0,19585 | -0,2671 | -0,07795 | -0,06083 | -0,05582 | -0,13 |
| **Level of significance** | 0,170193 | 0,766936 | 0,964511 | 0,418889 | 0,566315 | 0,916126 | 0,880188 | 0,41408 | 0,82737 | 0,166086 | 0,065859 | 0,011395 | 0,467802 | 0,577926 | 0,609749 | 0,23287 |
| **Serum IL-7** | -0,06101 | -0,04493 | 0,186366 | -0,12167 | -0,03393 | -0,22059 | -0,16231 | -0,19261 | 0,009104 | -0,02019 | -0,25736 | -0,17161 | -0,08647 | 0,047333 | 0,166745 | 0,098751 |
| **Level of significance** | 0,570058 | 0,675887 | 0,080347 | 0,256013 | 0,75223 | 0,037777 | 0,128595 | 0,070545 | 0,932518 | 0,868222 | 0,014903 | 0,107819 | 0,420414 | 0,665183 | 0,124914 | 0,36568 |
| **IL-7 ratio** | -0,06144 | 0,035565 | -0,11604 | 0,01202 | 0,0541 | 0,138607 | 0,103335 | 0,042454 | -0,0106 | -0,09419 | 0,048945 | -0,08199 | -0,00289 | -0,08896 | -0,15021 | -0,16739 |
| **Level of significance** | 0,567341 | 0,740735 | 0,278861 | 0,910981 | 0,614589 | 0,195186 | 0,335215 | 0,692826 | 0,921477 | 0,437993 | 0,648759 | 0,444967 | 0,978526 | 0,415357 | 0,167461 | 0,123451 |
| **CSF IL-12/IL-23p40** | 0,010642 | -0,05425 | 0,021059 | -0,00506 | -0,02017 | -0,05851 | 0,021173 | -0,0538 | -0,01335 | 0,259038 | 0,173971 | 0,177487 | -0,14494 | 0,126441 | 0,089439 | -0,0543 |
| **Level of significance** | 0,921157 | 0,613631 | 0,844701 | 0,962457 | 0,851176 | 0,586006 | 0,843873 | 0,616575 | 0,901197 | 0,030361 | 0,102997 | 0,096117 | 0,175366 | 0,24602 | 0,412829 | 0,619531 |
| **Serum IL12/IL23p40** | -0,13183 | -0,07932 | 0,214123 | -0,10955 | -0,05298 | -0,10379 | -0,12756 | -0,22412 | 0,108366 | -0,1427 | -0,11518 | -0,05881 | -0,14236 | -0,15356 | -0,07095 | -0,14621 |
| **Level of significance** | 0,218134 | 0,459959 | 0,043914 | 0,306778 | 0,621942 | 0,333093 | 0,233534 | 0,034736 | 0,312087 | 0,238602 | 0,282463 | 0,584071 | 0,183257 | 0,158077 | 0,516256 | 0,17919 |
| **IL-12/IL-23p40 ratio** | 0,120803 | -0,01516 | -0,07135 | 0,068104 | 0,01788 | 0,033824 | 0,090225 | 0,09016 | -0,0658 | 0,300137 | 0,246731 | 0,198273 | -0,04365 | 0,196954 | 0,11509 | 0,030888 |
| **Level of significance** | 0,259456 | 0,88788 | 0,506413 | 0,525984 | 0,867914 | 0,753011 | 0,400426 | 0,400767 | 0,540095 | 0,011589 | 0,019758 | 0,062519 | 0,684621 | 0,069126 | 0,291335 | 0,777696 |
| **CSF IL-15** | -0,10185 | -0,0267 | -0,01332 | -0,09751 | -0,09727 | -0,05939 | -0,04788 | -0,22403 | -0,00279 | -0,03679 | 0,045574 | 0,08834 | 0,013092 | 0,10227 | -0,01168 | -0,00189 |
| **Level of significance** | 0,342246 | 0,803881 | 0,901422 | 0,363315 | 0,364484 | 0,580364 | 0,655939 | 0,034816 | 0,979314 | 0,762373 | 0,671501 | 0,410379 | 0,903084 | 0,348762 | 0,915001 | 0,986209 |
| **Serum IL-15** | 0,112569 | -0,01825 | 0,178103 | -0,00622 | -0,10261 | -0,15075 | -0,07733 | -0,06051 | 0,093151 | -0,12619 | -0,19409 | -0,09711 | 0,050027 | 0,103932 | 0,118347 | 0,124782 |
| **Level of significance** | 0,293579 | 0,865196 | 0,09495 | 0,953844 | 0,338643 | 0,158487 | 0,471351 | 0,573256 | 0,385254 | 0,297908 | 0,068377 | 0,365301 | 0,641523 | 0,340943 | 0,277803 | 0,25232 |
| **IL-15 ratio** | -0,12286 | 0,015993 | -0,16901 | -0,04104 | 0,047416 | 0,145 | 0,08819 | -0,12247 | -0,04849 | 0,0965 | 0,154801 | 0,156419 | -0,02596 | 0,017388 | -0,09415 | -0,0977 |
| **Level of significance** | 0,251359 | 0,881747 | 0,113355 | 0,702557 | 0,659032 | 0,175173 | 0,411178 | 0,252907 | 0,651821 | 0,426792 | 0,14748 | 0,143244 | 0,809166 | 0,873748 | 0,388569 | 0,370815 |
| **CSF IL-17A** | 0,074511 | 0,030312 | -0,09062 | 0,021971 | -0,00676 | -0,07456 | -0,00216 | -0,02283 | -0,0064 | 0,195056 | 0,148601 | 0,063399 | -0,06321 | 0,230775 | 0,149248 | -0,08133 |
| **Level of significance** | 0,487706 | 0,777953 | 0,398372 | 0,838064 | 0,949857 | 0,487424 | 0,983983 | 0,83179 | 0,952547 | 0,105629 | 0,164586 | 0,555024 | 0,556197 | 0,032537 | 0,170216 | 0,45663 |
| **Serum IL-17A** | 0,067504 | -0,24505 | -0,07723 | -0,09303 | -0,09559 | -0,23351 | -0,20094 | -0,11541 | 0,03031 | 0,030692 | 0,042545 | 0,016429 | 0,041873 | 0,072365 | 0,132257 | 0,051198 |
| **Level of significance** | 0,529645 | 0,02064 | 0,471922 | 0,3859 | 0,372882 | 0,027645 | 0,058997 | 0,281473 | 0,777966 | 0,800867 | 0,692198 | 0,878548 | 0,696824 | 0,507885 | 0,22479 | 0,639677 |
| **IL-17A ratio** | 0,03464 | 0,185246 | 0,006171 | 0,118357 | 0,069869 | 0,116148 | 0,155837 | 0,080847 | 0,015008 | 0,187032 | 0,138662 | 0,094502 | -0,07312 | 0,204775 | 0,087448 | -0,07921 |
| **Level of significance** | 0,747245 | 0,082215 | 0,954229 | 0,269299 | 0,515294 | 0,278399 | 0,144756 | 0,451356 | 0,888984 | 0,121054 | 0,195006 | 0,378374 | 0,495895 | 0,058583 | 0,42335 | 0,468471 |
| **CSF MIP-1b** | -0,01523 | -0,0574 | 0,007686 | 0,040275 | 0,04075 | 0,0063 | 0,007534 | -0,06036 | 0,168848 | 0,092143 | -0,06544 | -0,01559 | -0,08784 | 0,085844 | 0,029011 | 0,064551 |
| **Level of significance** | 0,88735 | 0,593116 | 0,943008 | 0,707857 | 0,704573 | 0,953276 | 0,944134 | 0,57421 | 0,113702 | 0,448058 | 0,542329 | 0,884676 | 0,413018 | 0,431933 | 0,790891 | 0,554873 |
| **Serum MIP-1b** | 0,016788 | -0,01286 | -0,08533 | -0,02299 | -0,05037 | -0,06248 | -0,09003 | -0,09122 | 0,268063 | -0,06138 | -0,13241 | -0,09591 | -0,11279 | -0,16721 | -0,17209 | -0,08852 |
| **Level of significance** | 0,875914 | 0,904809 | 0,426595 | 0,830638 | 0,639229 | 0,560789 | 0,401453 | 0,395208 | 0,011091 | 0,613709 | 0,216092 | 0,371296 | 0,292647 | 0,123859 | 0,113099 | 0,417645 |
| **MIP-1b ratio** | -0,04631 | -0,05501 | 0,033265 | 0,059132 | 0,104164 | 0,091417 | 0,080596 | 0,031072 | -0,14215 | 0,123219 | 0,057354 | 0,085836 | 0,100596 | 0,243248 | 0,23719 | 0,221493 |
| **Level of significance** | 0,666496 | 0,608648 | 0,756961 | 0,582017 | 0,33133 | 0,394204 | 0,452764 | 0,772537 | 0,183893 | 0,309517 | 0,59343 | 0,423822 | 0,348252 | 0,024021 | 0,027884 | 0,040412 |
| **CSF MCP-1** | -0,10011 | -0,13634 | 0,05824 | 0,043902 | 0,014296 | 0,219909 | -0,03746 | -0,05925 | 0,137683 | 0,068206 | -0,09086 | -0,0498 | -0,06147 | -0,04654 | -0,05701 | -0,17534 |
| **Level of significance** | 0,35061 | 0,202669 | 0,58773 | 0,682899 | 0,894222 | 0,038385 | 0,727456 | 0,581263 | 0,198207 | 0,574777 | 0,397113 | 0,643006 | 0,567125 | 0,670464 | 0,602091 | 0,10636 |
| **Serum MCP-1** | 0,0339 | -0,01785 | -0,1398 | -0,02196 | -0,07355 | 0,042269 | -0,09726 | -0,05044 | 0,260743 | 0,019178 | -0,12314 | -0,17088 | -0,20287 | -0,16542 | -0,1493 | -0,22284 |
| **Level of significance** | 0,752473 | 0,868098 | 0,191319 | 0,83818 | 0,493343 | 0,694094 | 0,364564 | 0,638747 | 0,01359 | 0,87479 | 0,250298 | 0,109354 | 0,056561 | 0,127973 | 0,170065 | 0,039176 |
| **MCP1 ratio** | -0,16677 | -0,13772 | 0,133802 | -0,05466 | -0,01634 | 0,06523 | -0,00779 | -0,07074 | -0,15824 | 0,013403 | 0,050426 | 0,09205 | 0,130541 | 0,101118 | 0,06856 | 0,017312 |
| **Level of significance** | 0,118291 | 0,198092 | 0,211273 | 0,610912 | 0,879208 | 0,543637 | 0,942244 | 0,510045 | 0,138582 | 0,91231 | 0,638864 | 0,390922 | 0,222715 | 0,354244 | 0,530507 | 0,874292 |
| **CSF MCP4** | 0,276723 | 0,087546 | -0,0694 | 0,26142 | 0,12365 | 0,270412 | 0,164036 | 0,220755 | -0,05871 | 0,097914 | 0,093451 | 0,17469 | 0,176456 | 0,017733 | 0,064751 | 0,085928 |
| **Level of significance** | 0,008661 | 0,414617 | 0,518094 | 0,01334 | 0,248311 | 0,010379 | 0,124524 | 0,037629 | 0,584693 | 0,420014 | 0,383724 | 0,10156 | 0,098097 | 0,87126 | 0,553645 | 0,431481 |
| **Serum MCP-4** | -0,02197 | -0,03511 | -0,18368 | -0,09708 | -0,17735 | -0,09128 | -0,15281 | -0,11445 | 0,335467 | 0,064392 | -0,13498 | -0,15919 | -0,11571 | -0,10502 | -0,13235 | -0,0718 |
| **Level of significance** | 0,838084 | 0,743912 | 0,084876 | 0,365448 | 0,09637 | 0,394932 | 0,152818 | 0,285528 | 0,00131 | 0,596395 | 0,207256 | 0,136191 | 0,280248 | 0,335906 | 0,224472 | 0,511233 |
| **MCP-4 ratio** | 0,254793 | 0,046386 | -0,00113 | 0,24933 | 0,140137 | 0,262417 | 0,19144 | 0,224016 | -0,16818 | 0,098214 | 0,154086 | 0,220089 | 0,221025 | 0,089825 | 0,135167 | 0,119601 |
| **Level of significance** | 0,015968 | 0,665995 | 0,991599 | 0,01846 | 0,19025 | 0,012979 | 0,072309 | 0,034825 | 0,115169 | 0,418582 | 0,149382 | 0,038223 | 0,03739 | 0,410803 | 0,214663 | 0,27271 |
| **CSF VEGF** | 0,053929 | 0,148371 | 0,004741 | 0,011736 | -0,02626 | -0,21974 | -0,095 | -0,04963 | -0,03612 | -0,05593 | -0,00234 | 0,029189 | -0,00458 | 0,098689 | 0,069639 | 0,048326 |
| **Level of significance** | 0,615708 | 0,165248 | 0,964826 | 0,913081 | 0,807022 | 0,038535 | 0,375873 | 0,644158 | 0,7368 | 0,645584 | 0,98263 | 0,785978 | 0,966025 | 0,365984 | 0,524043 | 0,658596 |
| **Serum VEGF** | 0,116334 | -0,15022 | 0,039215 | -0,07807 | -0,16743 | -0,06109 | -0,21854 | -0,13931 | -0,11836 | -0,15487 | 0,061015 | -0,0248 | 0,029094 | 0,062588 | 0,043634 | 0,105232 |
| **Level of significance** | 0,277627 | 0,15999 | 0,71521 | 0,46708 | 0,116821 | 0,569542 | 0,03964 | 0,192898 | 0,269287 | 0,200489 | 0,570033 | 0,817524 | 0,786658 | 0,566992 | 0,689956 | 0,334905 |
| **VEGF ratio** | -0,10085 | 0,176666 | -0,04419 | 0,075749 | 0,135821 | -0,00223 | 0,180636 | 0,119444 | 0,089808 | 0,111355 | -0,05553 | 0,036611 | -0,02981 | -0,0553 | -0,05011 | -0,09201 |
| **Level of significance** | 0,347039 | 0,097691 | 0,680896 | 0,480483 | 0,204403 | 0,983449 | 0,090266 | 0,264894 | 0,402612 | 0,358751 | 0,605234 | 0,733393 | 0,781545 | 0,613087 | 0,646842 | 0,399448 |

**Supplementary table I8. Correlation between cell surface marker expression and soluble markers of activation and inflammation in serum and CSF, and their respective ratios.** Calculations were performed using Pearson correlations. % of CD11b, CD18, CD40, CD64, CD86, CD163, CCR1, CCR2, CCR5, and TACE positive monocytes, and HERV H3 Env and HERV W3 Env expression on each of the three monocyte subsets (classical, intermediate, and non-classical monocytes); IFNγ (interferon gamma), IL-1β (interleukin-1β), IL-2 (interleukin-2), IL-6 (interleukin-6), IL-8 (interleukin-8), IL-10 (interleukin-10), TNFα (tumor necrosis factor alpha), GMCSF (granulocyte-macrophage colony stimulating factor), IL-5 (interleukin-5), IL-7 (interleukin-7), IL-12/IL-23p40 (interleukin-12/interleukin 23 p40), IL-15 (interleukin-15), IL-17A (interleukin-17A), VEGF (vascular endothelial factor), MIP-1β (macrophage inflammatory protein-1β), MCP-1 (monocyte chemoattractant protein-1), MCP-4 (monocyte chemoattractant protein-4), CIS (clinically isolated syndrome), RRMS (relapsing-remitting MS), PMS (progressive MS), RIS (radiologically isolated syndrome), SC (symptomatic controls), n (number of subjects).

**Supplementary figure I3**


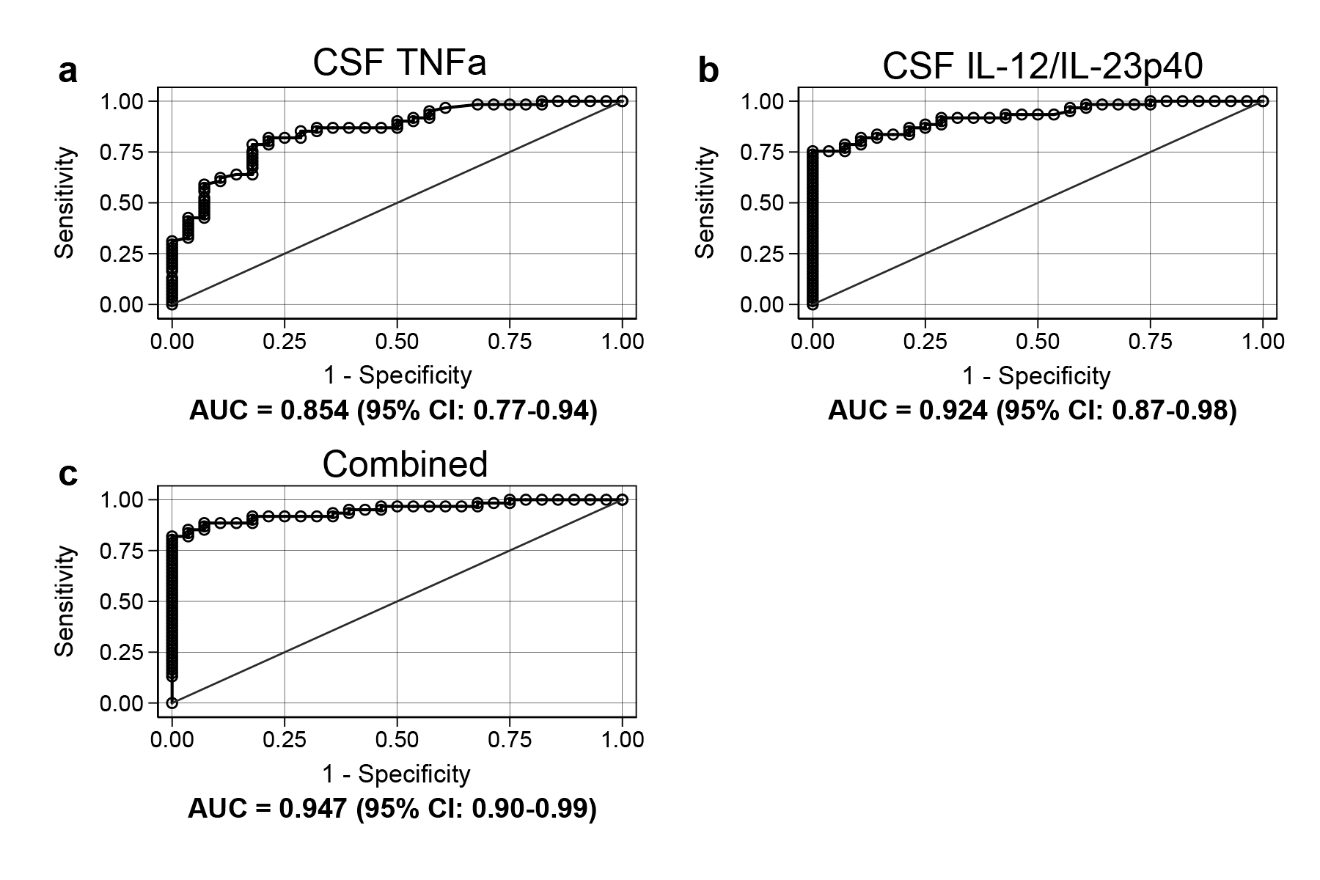


**Supplementary figure I3. Logistic regression analysis with ROC curve output of the two soluble markers with the highest AUC identified from Figure 6, individually, and combined.** AUC with 95% CI is given for each parameter. The soluble marker values of each parameter for patients (CIS + RRMS + PMS) are combined as true positives and plotted against controls (SC + RIS) as true negatives for TNFα **(a)** and IL-12/IL-23p40 **(b)** in CSF. A logistic regression analysis with the combined parameter results was also performed **(c)**. The diagonal dividing the ROC space represents the random event. ROC (receiver operating characteristic), AUC (area under the curve), CIS (clinically isolated syndrome), RRMS (relapsing-remitting MS), PMS (progressive MS), RIS (radiologically isolated syndrome), and SC (symptomatic controls).

**Supplementary figure I4**


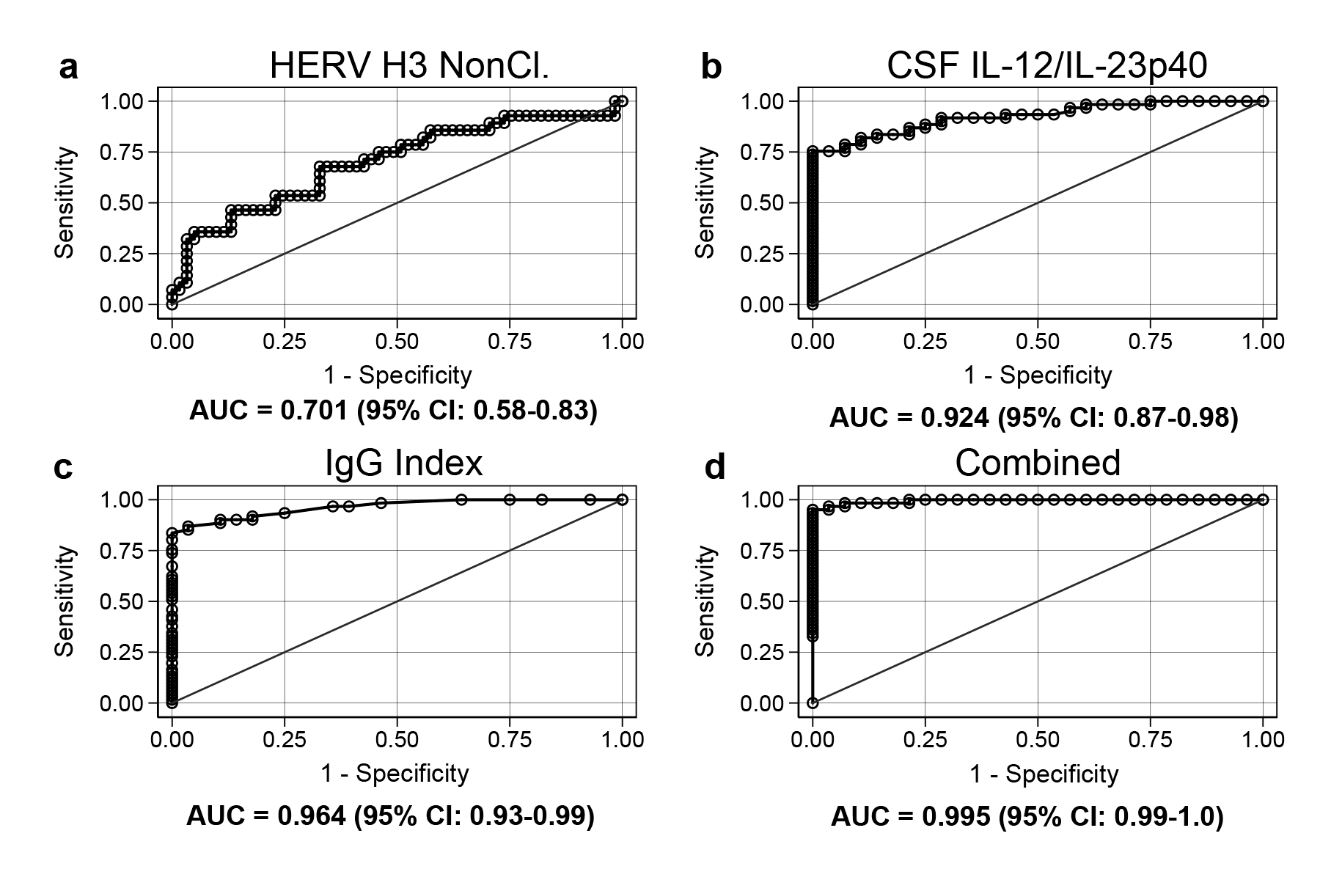


**Supplementary figure I4. Logistic regression analysis with ROC curve output of the soluble markers with the highest AUC as well as HERV H3 Env, individually, and combined.** AUC with 95% CI is given for each parameter. The soluble marker values of each parameter for patients (CIS + RRMS + PMS) are combined as true positives and plotted against controls (SC + RIS) as true negatives for HERV H3 Env on the non-classical monocyte population **(a)** and IL-12/IL-23p40 in CSF **(b)** as well as for the IgG Index **(c)**. A logistic regression analysis with the combined parameter results was also performed **(d)**. The diagonal dividing the ROC space represents the random event. ROC (receiver operating characteristic), AUC (area under the curve), CIS (clinically isolated syndrome), RRMS (relapsing-remitting MS), PMS (progressive MS), RIS (radiologically isolated syndrome), and SC (symptomatic controls).

**Supplementary figure I5**

**
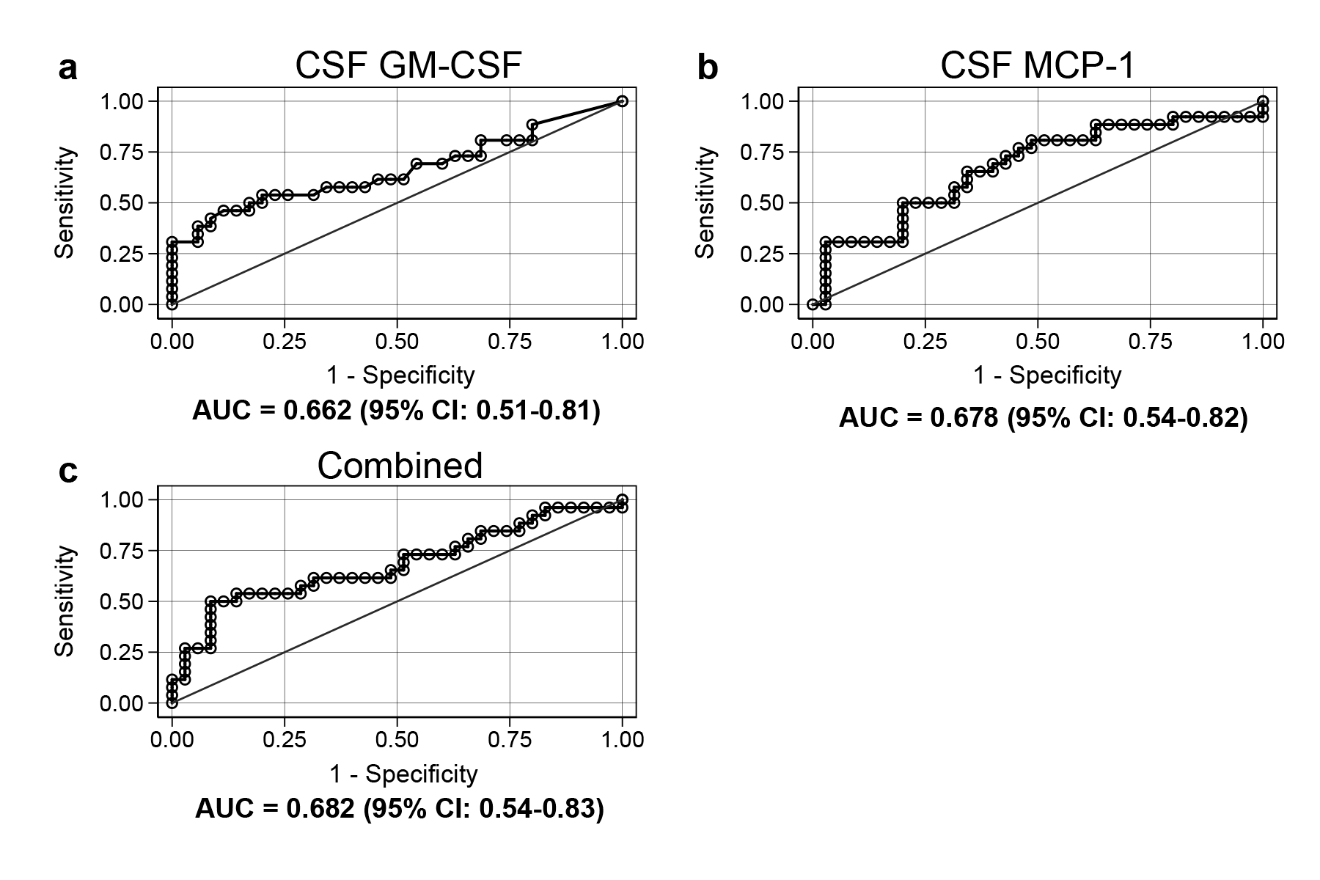
**

**Supplementary figure I5. Logistic regression analysis with ROC curve output of the two soluble markers that were significantly different in Supplementary table I5 between patients that progressed and patients that did not, individually, and combined.** AUC with 95% CI is given for each parameter. The soluble marker values of each parameter for patients (CIS + RRMS + PMS) that progressed are combined as true positives and plotted against patients (CIS + RRMS + PMS) that did not progress as true negatives for GM-CSF **(a)** and MCP-1 **(b)** in CSF. A logistic regression analysis with the combined parameter results was also performed **(c)**. The diagonal dividing the ROC space represents the random event. ROC (receiver operating characteristic), AUC (area under the curve), CIS (clinically isolated syndrome), RRMS (relapsing-remitting MS), PMS (progressive MS).

**Supplementary figure I6**

**
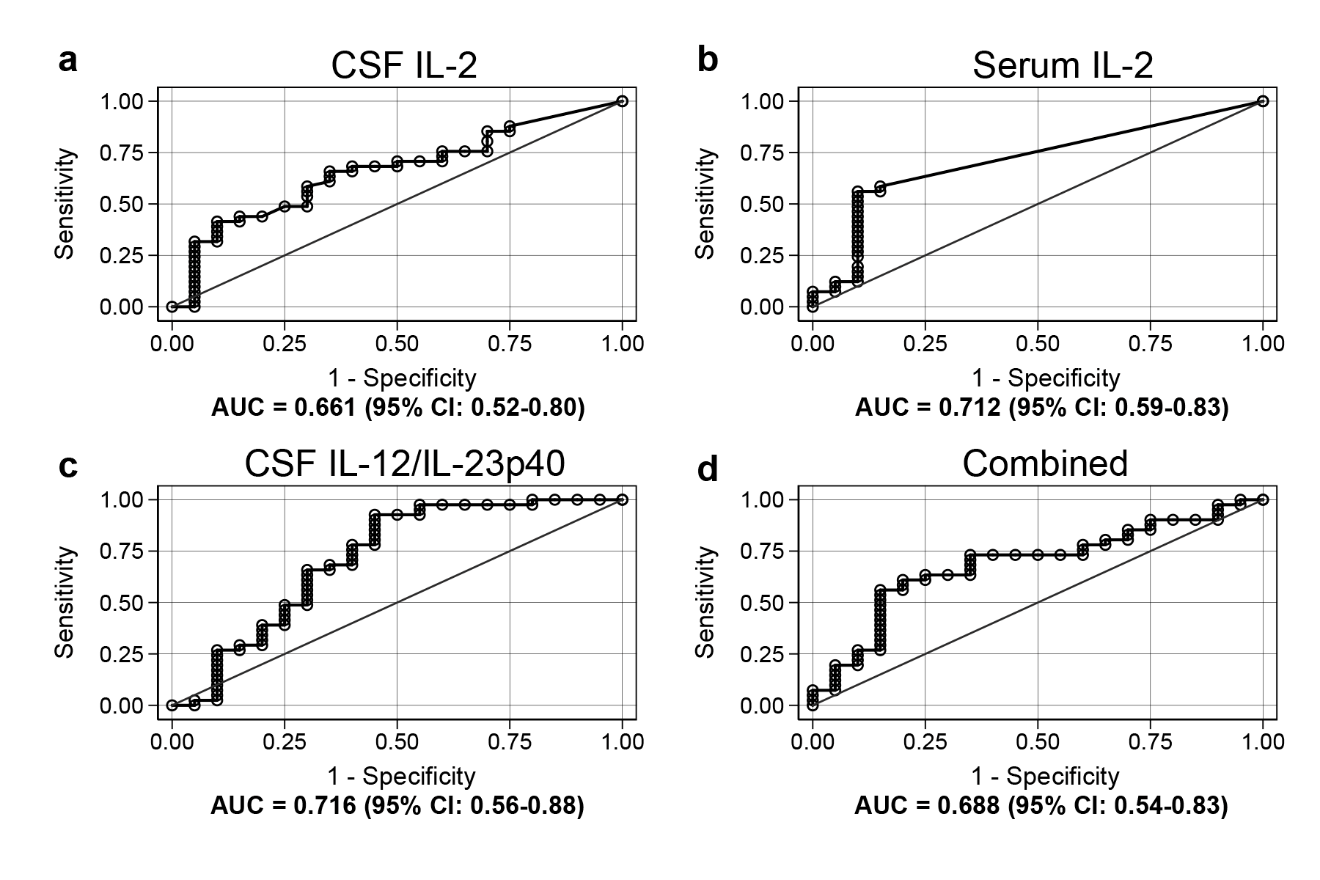
**

**Supplementary figure I6. Logistic regression analysis with ROC curve output of the three soluble markers that were significantly different in Supplementary table I5 and Supplementary table I6 between patients that receive medication and patients that did not, individually, and combined.** AUC with 95% CI is given for each parameter. The soluble marker values of each parameter for patients (CIS + RRMS + PMS) that received medication are combined as true positives and plotted against patients (CIS + RRMS + PMS) that did not as true negatives for IL-2 **(a)** in CSF, IL-2 in serum **(b)**, IL-12/IL-23p40 **(c)** in CSF. A logistic regression analysis with the combined parameter results was also performed **(d)**. The diagonal dividing the ROC space represents the random event. Abbreviations: ROC (receiver operating characteristic), AUC (area under the curve), CIS (clinically isolated syndrome), RRMS (relapsing-remitting MS), PMS (progressive MS).

| **Supplementary table I9. Symptomatic controls and radiologically isolated syndrome** |
| --- |
| #5: 43-year-old woman with paresthesia in the hands and numbness of the right foot during the last year. Sensation of weakness of this foot when playing tennis. On examination (OE) slight dysesthesia on the right side of the right leg. No hard neurological signs. Treated with TNF-alpha blocking agents and azathioprine during the last years for Crohn. Cortical T2-weighted/T2- fluid attenuated inversion recovery (FLAIR) MRI showed nonspecific White Matter (WM) lesions, and normal MRI of the spinal cord. Spinal fluid normal, and visual and somatosensory evoked potentials normal. Diagnosis: Paresthesia. |
| #11: 41-year-old woman with paresthesia in the right arm and leg during the last couple of years. Could not walk for as long as previously and experienced reduced power in the right arm. Otherwise no medical history. OE normal neurological findings. Cortical Flair MRI showed nonspecific WM lesions but also four periventricular WM lesions suggestive of MS. Normal MRI of the spinal cord, spinal fluid and evoked potentials. With no typical relapsing-remitting or progressive symptoms and no documented objective neurological findings the diagnosis was: Radiologically isolated syndrome. |
| #14: 22-year-old woman reporting sudden dizziness after raising her head followed by blurred vision. Asthma, otherwise no medical history. Normal otological examination. OE slight reduction in visual acuity on the left eye. Cortical and spinal cord Flair MRI normal as well as spinal fluid and visual evoked potentials. Diagnosis: Unspecific visual disturbances. |
| #16: 46-year-old man with sudden paresthesia in the right arm followed by dizziness in relation to fast movements of the head. Medical history with psoriatic arthropathy for 20 years treated with TNF-alpha blocking agents and methotrexate. OE normal neurological findings. Cortical Flair MRI showed nonspecific WM lesions and normal MRI of the spinal cord, spinal fluid and evoked potentials. With no typical relapsing-remitting or progressive symptoms and no documented objective neurological findings the diagnosis was: Nonspecific dizziness. |
| #19: 20-year-old woman with back pain during five years and during the last year paresthesia in the arms and legs in relation to physical activity. OE normal neurological findings. Cortical and spinal cord Flair MRI normal as well as spinal fluid and evoked potentials. The diagnosis was: Back pain. |
| #20: 59-year-old woman with paresthesia in arms and legs during the last couple of years. In addition during eight years blurred vision on the right eye lasting for weeks. No medical history otherwise. Treated for two years with azathioprine. OE reduced vision to one third on the left eye otherwise normal neurological findings. Cortical Flair MRI and spinal cord normal as well as spinal fluid and evoked potentials and no aquaporin-4 antibodies. With no documented objective neurological findings the diagnosis was: Unspecific visual disturbances. |
| #21: 56-year-old woman with remitting warm sensation and tenderness for weeks in the right sided extremities for three years. Treated with eltroxin for thyroid disorder. OE normal neurological findings. Cortical and spinal cord Flair MRI normal as well as spinal fluid and evoked potentials. With no documented objective neurological findings the diagnosis was: Paresthesia. |
| #27: 44-year-old man with sudden onset of reduced sensation in the right leg sustained for a week. Medical history migraine with aura. OE normal neurological findings. Cortical Flair MRI showed nonspecific WM lesions and normal MRI of the spinal cord, spinal fluid and evoked potentials. With no documented objective neurological findings the diagnosis was: Paresthesia. |
| #31: 56-year-old woman reporting sudden dizziness after raising her head. The dizziness lasting for a month and was accompanied with tension headache. No medical history. OE normal neurological findings. Cortical Flair MRI showed nonspecific WM lesions, and normal MRI of the spinal cord. Spinal fluid normal and evoked potentials normal. Diagnosis: Nonspecific dizziness. |
| #34: 35-year-old woman with dizziness and tension headache after influenza three months previously. Normal otological examination. OE normal neurological findings. Cortical Flair MRI showed nonspecific WM lesions, and normal MRI of the spinal cord. Spinal fluid and evoked potentials normal. Diagnosis: Nonspecific dizziness. |
| #40: 44-year-old woman who woke with dizziness, fluctuating the following weeks. Previously a period with paresthesia in the left hand two years back. Born with a slight strabismus. Normal otological examination. OE slight reduced visual acuity on the left side and slight unsteadiness at Romberg. Cortical flair MRI showed nonspecific WM lesions, and normal MRI of the spinal cord.Spinal fluid normal. Diagnosis: Nonspecific dizziness. |
| #41: 40-year-old woman examined because of lactorrhea during a month. In addition, periods of pain and disturbances of sensation in arms and legs for days in the last three years. For many years migraine with aura. The last year experienced fatique both physically and mentally. OE normal neurological findings. Cortical Flair MRI showed nonspecific WM lesions but also one periventricular WM lesion suggestive of MS. Normal MRI of the spinal cord, spinal fluid and evoked potentials. With no typical relapsing-remitting or progressive symptoms and no documented objective neurological findings the diagnosis was: Radiologically isolated syndrome. |
| #42: 35-year-old woman with sudden pain in the left hand followed by tiredness of the hand during work for a week, and slight disturbances of the balance. One year previously just after delivery she experienced reduced power during lift of the right foot for weeks. Finally, an episode with general unease and problems with focusing and expressing herself for 10 minutes two weeks ago. OE slight unsteadiness at Romberg and reduced sensation in the right foot but no hard neurological signs. Cortical Flair MRI showed nonspecific WM lesions but also three periventricular WM lesions suggestive of MS. Normal MRI of the spinal cord, spinal fluid and evoked potentials. With no typical relapsing-remitting or progressive symptoms and no documented objective neurological findings the diagnosis was: Radiologically isolated syndrome. |
| #44: 31-year-old man with progressive sensory disturbances in the left sided extremities lasting for two weeks. No medical history. OE normal neurological findings. Cortical Flair MRI showed nonspecific WM lesions, and normal MRI of the spinal cord and spinal fluid. With no documented objective neurological findings the diagnosis was: Paresthesia. |
| #46: 38-year-old man with sensory disturbances in the right side of the face and shortly after also in the right sided extremities lasting for a week. Previously a year back examined because of sudden onset of breast pain. OE normal neurological findings. Cortical Flair MRI showed nonspecific WM lesions, normal MRI of the spinal cord, spinal fluid and evoked potentials. With no documented objective neurological findings the diagnosis was: Paresthesia. |
| #49: 39-year-old woman with increasing sensory disturbances and dysesthesia in the left side of the face for two years. Five years previously operated for thyroid disease, no need for medication afterwards. OE dysesthesia in the left side of the face. Cortical Flair MRI showed nonspecific WM lesions, normal MRI of the spinal cord, spinal fluid and evoked potentials. With no documented objective neurological findings the diagnosis was: Dysesthesia. |
| #52: 28-year-old woman with visual disturbances on both eyes lasting for weeks. For months registered reduced accuracy of the arms during daily activity. Same period urgency and pain at the backside of the left leg. Otherwise no medical history. OE dysesthesia slight reduction of acuity on the left eye. Cortical and spinal cord flair MRI normal, normal spinal fluid and evoked potentials. With no documented objective neurological findings the diagnosis was: Unspecific visual disturbances. |
| #53: 36-year-old woman with periodic paresthesia in arms and legs for three months. No previous medical history. OE normal neurological findings. Cortical and spinal cord flair MRI normal, normal spinal fluid and neurography. With no documented objective neurological findings the diagnosis was: Paresthesia. |
| #54: 38-year-old woman with dizziness after influenza. Later difficulties with coordination of the left arm and sensory disturbances in the left side of the face and left arm. Normal otological and neurological examination. Cortical Flair MRI showed nonspecific WM lesions, normal MRI of the spinal cord and spinal fluid normal. Diagnosis: Nonspecific dizziness. |
| #60: 21-year-old woman with progressive visual disturbances on the left eye for four weeks. In the same period registered Lhermitte’s sign . For some years registered reduced memory and treated with sertraline for depression. Otherwise no medical history. OE slight uncertainty during coordination tests on the left arm, reduced sensation of vibration on both feet and unsteadiness at Rombergs test but not on repetition. Cortical and spinal cord flair MRI normal, normal spinal fluid and evoked potentials. With no documented objective neurological findings the diagnosis was: Unspecific visual disturbances. |
| #61: 25-year-old woman with back pain and reduced power in the legs, slightly improved during the last weeks. Medical history: tendency to herpes reactivations. OE unsteadiness at Rombergs and slightly reduced strength in the left leg. Cortical Flair MRI showed nonspecific WM lesions, normal MRI of the spinal cord as well as spinal fluid and evoked potentials. The diagnosis was: Back pain. |
| #62: 44-year-old woman with pulse synchronic tinnitus followed by sudden deafness on the left side. Some days after that she registered reduced vision on the left eye. OE visual acuity on the left side 0.5, perceptic hearing loss on the left side. Cortical Flair MRI showed nonspecific WM lesions but also five periventricular WM lesions suggestive of MS. Normal MRI of the spinal cord, spinal fluid and evoked potentials. With atypical hearing loss and otherwise no documented objective neurological findings the diagnosis was: Radiologically isolated syndrome. |
| #66: 51-year-old woman with periodic symptoms for 10 years lasting four to five months. The symptoms could be balance problems during walking or during standing and reduced gait distance, blurred vision and urgency, sensory disturbances in the legs and fatique. OE normal neurological findings. Cortical Flair MRI showed nonspecific WM lesions but also eight periventricular WM lesions suggestive of MS. Normal MRI of the spinal cord, spinal fluid and evoked potentials. With no documented objective neurological findings the diagnosis was: Radiologically isolated syndrome. |
| #73: 23-year-old woman with back pain two months back followed by paresthesia in the left arm. For some years urgency otherwise no previous medical history. OE normal neurological findings. Cortical Flair MRI showed nonspecific WM lesions, normal MRI of the spinal cord as well as spinal fluid and evoked potentials. With no documented objective neurological findings the diagnosis was: Paresthesia. |
| #77: 48-year-old woman with a burning sensation in the legs after an infection. During the last weeks sudden neuralgic pain in the left side of the face. Ten years ago lumbar disc operation otherwise no previous medical history. OE reduced Achilles reflex on the left side otherwise normal neurological findings. Cortical and spinal cord flair MRI normal, normal spinal fluid and evoked potentials. With no documented objective neurological findings the diagnosis was: Paresthesia. |
| #78: 33-year-old woman with sensory disturbances in arms and legs for four months, the last two combined with a weakness in the right leg. Lately she experienced hypersensitivity for sounds and reduced cognitive function. Diagnosed with spondyloarthritis and treated with NSAID. Otherwise no medical history. OE slight reduced power in hip flexion but accompanied with increased pain in the hip. Cortical and spinal cord flair MRI normal, and normal spinal fluids. With no definite documented objective neurological findings the diagnosis was: Paresthesia. |
| #79: 24-year-old woman with paresthesia in the arms for a year with reduced power in the left arm. No medical history. Cortical and spinal cord flair MRI normal, neurography and normal spinal fluids. With no documented objective neurological findings the diagnosis was: Paresthesia. |
| #84: 42-year-old woman with paresthesia in periods in the legs and tension headache in the last two years. In addition two episodes of blurred vision lasting for days and provoked by sudden rise from sitting position. No medical history otherwise. OE normal neurological findings. Cortical Flair MRI showed nonspecific WM lesions but also four periventricular WM lesions suggestive of MS. Normal MRI of the spinal cord, spinal fluid and evoked potentials. With atypical symptoms and no documented objective neurological findings the diagnosis was: Radiologically isolated syndrome. |
